# Regulatory Effect of PGE_2_-EP2/EP4 Receptor Pathway on *Staphylococcus aureus*-Induced Inflammatory Factors in Dairy Cow Neutrophils

**DOI:** 10.3390/biom15081062

**Published:** 2025-07-22

**Authors:** Yi Zhao, Chao Wang, Bo Liu, Shuangyi Zhang, Yongfei Wang, Yinghong Qian, Zhiguo Gong, Jiamin Zhao, Xiaolin Yang, Yuting Bai, Wei Mao

**Affiliations:** 1 Laboratory of Veterinary Clinical Pharmacology, College of Veterinary Medicine, Inner Mongolia Agricultural University, No. 29, Erdosdong Road, Saihan District, Hohhot 010011, China; zoey18047287583@163.com (Y.Z.);; 2 Key Laboratory of Clinical Diagnosis and Treatment Techniques for Animal Disease, Ministry of Agriculture, Inner Mongolia Agricultural University, No. 29, Erdosdong Road, Saihan District, Hohhot 010011, China; 3 Laboratory Animal Center, Inner Mongolia Medical University, Hohhot 010030, China; 4 Inner Mongolia Academy of Agricultural & Animal Husbandry Science, Hohhot 010010, China

**Keywords:** neutrophils of cows, *Staphylococcus aureus* Rosenbach, prostaglandin E_2_, EP2/EP4 receptor pathway, anti-inflammatory therapy

## Abstract

Naturally occurring prostaglandin E_2_ (PGE_2_) influences cytokine production regulation in bovine neutrophils exposed to *Staphylococcus aureus* Rosenbach. Here, we employed bovine neutrophils as the primary experimental system, and administered specific inhibitors targeting various receptors, which were subsequently exposed to *S. aureus*. Cytokine expression levels in dairy cow neutrophils induced by *S. aureus* via the endogenous PGE_2_-EP2/4 receptor pathway were investigated, and its effects on P38, extracellular signal-regulated kinase (ERK), P65 activation, and phagocytic function in *Staphylococcus aureus* Rosenbach-induced dairy cow neutrophils, were examined. Blocking cyclooxygenase-2 (COX-2) and microsomal prostaglandin E synthase-1 (mPGES-1) enzymes substantially decreased PGE_2_ production and release in *S. aureus*-exposed bovine neutrophils. Cytokine output showed significant reduction compared to that in *SA113*-infected controls. Phosphorylation of P38, ERK, and P65 signaling molecules was depressed in the infected group. Pharmacological interference with EP2/EP4 receptors similarly diminished cytokine secretion and phosphorylation patterns of P38, ERK, and P65, with preserved cellular phagocytic function. During *S. aureus* infection of bovine neutrophils, COX-2 and mPGES-1 participated in controlling PGE_2_ biosynthesis, and internally produced PGE_2_ molecules triggered NF-κB and MAPK inflammatory pathways via EP2/EP4 receptor activation, later adjusting the equilibrium between cytokine types that promote or suppress inflammation. This signaling mechanism coordinated inflammatory phases through receptor-mediated processes.

## 1. Introduction

Inflammation is an essential biological mechanism that preserves tissue equilibrium after physical damage or microbial invasion, facilitating the removal of harmful microorganisms while triggering restoration and recovery processes. In veterinary clinical practice, infections caused by *Staphylococcus aureus* Rosenbach (*S. aureus*) pose a significant challenge to the animal husbandry industry, being among the primary pathogenic bacteria responsible for postpartum diseases in dairy cows, including vaginitis and mastitis, and frequently coexist with multiple related pathological conditions [1,2]. Despite *S. aureus* employing complex strategies to circumvent immune responses, the host retains sufficient capacity to combat serious infections caused by this pathogen. This defense ability stems mainly from the pivotal function of neutrophils within the immunological protection framework of an organism. The local infiltration of neutrophils serves not only as a hallmark of *S. aureus* infection but also as an indicator of endometritis in dairy cows. Bhattacharya et al. (2020) observed that neutrophils displayed a remarkable capacity to rapidly phagocytose *S. aureus* [3]. Furthermore, the phagocytic capacity of *S. aureus* is significantly higher than that of *Klebsiella pneumoniae* Trevisan, *Streptococcus pyogenes* Rosenbach, and *Streptococcus pneumoniae* Fraenkel [4,5,6]. However, emerging research has revealed that exosomal prostaglandin E2 (PGE_2_) originating from M2 macrophages actively modulates neutrophil activity during sepsis through lipid mediator conversion mechanisms [7]. Experimental investigations using zebrafish inflammation models conducted by Loynes et al. established that blocking PGE_2_ production significantly delayed the resolution phase of neutrophil-mediated inflammatory processes [8]. Complementary findings demonstrate the capacity of exogenously added PGE_2_ to suppress phagocytic functions in human neutrophils via specific EP2 receptor-mediated signaling cascades involving the cAMP and PTEN pathways [9]. These collective observations strongly suggest the pivotal involvement of PGE_2_ in regulating inflammatory dynamics during *S. aureus* infection.

The production pathway for PGE_2_ initiates with arachidonic acid (AA) as the precursor molecule. Following phospholipase A2-mediated release of AA from membrane phospholipids, this precursor undergoes transformation into prostaglandin H_2_ (PGH_2_) and leukotrienes, subsequently being converted to PGE_2_. Prostaglandin E synthase-1 (PGES-1) facilitates the conversion process [10,11]. Research demonstrates that cyclooxygenase-2 (COX-2) and microsomal prostaglandin E synthase-1 (mPGES-1) operate as responsive enzymes essential for PGE_2_ formation under inflammatory conditions [12]. Evidence suggests that prostaglandins originating from COX-2 influence keratitis development by modulating inflammatory factors, including matrix metalloproteinase-1 and metalloproteinase-9 [13]. Clinically, non-steroidal anti-inflammatory medications (NSAIDs) and specific COX-2 blockers are frequently used to control inflammation. Their therapeutic action involves the inhibition of COX-1 or COX-2 functions, thereby reducing PGE_2_ synthesis [14]. However, their clinical application may lead to certain adverse effects, including gastric bleeding, ulcers, and perforation [15]. Moreover, they may also contribute to severe adverse reactions, including cardiovascular events such as heart attacks, cerebrovascular accidents, and elevated pulmonary arterial pressure [16]. Contemporary pharmacological investigations have increasingly focused on mPGES-1, another key enzyme in the PGE_2_ biosynthetic cascade [17]. Scientific evidence has revealed substantial overexpression of this protein in inflamed tissues and malignant growth, positioning it as a molecular bridge connecting inflammatory processes with oncogenesis, and as a viable therapeutic objective [18]. Additional research indicating that mPGES-1 is involved in multiple pathological conditions such as idiopathic joint degeneration, renal disorders, and cutaneous malignancies have been extensively documented [13,19,20]. Prior research conducted in our laboratory revealed that endometrial tissue infection by *Escherichia coli* Castellani & Chalmers (*E. coli*) in bovine subjects triggers substantial upregulation of both COX-2 and mPGES-1 enzymatic activity, concurrently elevating endogenous PGE_2_ production [21]. When evaluating therapeutic interventions, pharmacological agents targeting mPGES-1 demonstrated potentially superior safety characteristics compared to those of conventional COX-2 inhibiting anti-inflammatory compounds [22,23]. To date, various structurally diverse mPGES-1 inhibitors have been used in laboratory studies; however, these inhibitors display strong species specificity and are not broadly applicable to most animals. However, studies specifically targeting dairy cows are limited. Consequently, further experimental investigations and human trials are essential to determine if mPGES-1 suppression compounds demonstrate viable efficacy as prospective treatment options for inflammatory conditions and malignant growths [24,25,26].

PGE_2_ exerts diverse biological effects through its binding to four distinct receptor variants: EP1, EP2, EP3, and EP4. These molecular interactions mediate a wide range of physiological effects. These receptors display different functional properties and binding capacities for PGE_2_ in various physiological states. While EP3 and EP4 generally exhibit stronger PGE_2_ binding than EP1 and EP2, inflammatory processes and tumorigenesis trigger EP2/EP4-mediated activation of the cAMP/PKA cascade, influencing PGE_2_ biosynthesis and contributing to disease progression [27,28,29]. Qiu et al. (2023) revealed that EP2/EP4-dependent cAMP signaling serves as a crucial regulator of glioblastoma pathogenesis via PGE_2_ modulation [30]. Conventional NSAIDs inhibit PGE_2_ production by interfering with PGH_2_ metabolism, which inadvertently inhibits the production of prostaglandins that are essential for maintaining physiological homeostasis, leading to adverse side effects [31]. Alternatively, a selective EP4 receptor blocker was authorized for canine treatment, demonstrating significantly enhanced safety profiles compared to those of numerous existing pharmaceutical options [32]. This veterinary medication has fewer adverse effects than conventional therapies currently on the market [33].

In conclusion, this study hypothesis that PGE_2_ may regulate the inflammatory response triggered by *S. aureus* infection through its receptors EP2 and EP4. To investigate this hypothesis, bovine neutrophils were selected as experimental models. By inhibiting PGE_2_ synthesis or blocking its interaction with EP2/EP4 receptors, the regulatory role of the endogenous PGE_2_-EP2/EP4 signaling pathway in *S. aureus*-induced inflammatory responses in bovine neutrophils was examined. This study aimed to provide a theoretical foundation for the prevention and treatment of *S. aureus*-related diseases in cattle (including dairy cows) within veterinary clinical practice, as well as to offer scientific support for the development of novel therapeutic agents.

## 2. Materials and Methods

### 2.1. Materials

The healthy adult Holstein cows (n = 3) used in the experiment were all from the experimental base of Inner Mongolia Agricultural University. For specific individual information, please refer to Table S1. The reference strain, *S. aureus SA113* (ATCC 35556), was obtained from the Microbial Resource Center of the Chinese Academy of Sciences. The bovine neutrophil separation kit was purchased from Hyclone (Logan, UT, USA). The essential cell culture components included RPMI 1640 medium (HyClone, USA), fetal bovine serum (FBS; HyClone, USA), and a specialized bovine neutrophil isolation system (Tianjin Haoyang Biotech, Tianjin, China). The immunoassay reagents included a bovine IL-6 detection kit (R&D Systems, Minneapolis, MN, USA) and additional enzyme-linked immunosorbent assay (ELISA) testing materials (Kingfisher Biotech, Guangzhou, China). Electrophoresis supplies were procured from Thermo Fisher Scientific (Waltham, MA, USA). Protein analysis was performed using 10× Tris/glycine separation buffer (GENERAY, Shenzhen, China) and 10× loading buffer (Bio-Rad, Hercules, CA, USA).

#### 2.1.1. Antibodies and Molecular Reagents

Primary antibodies used included phospho-extracellular signal-regulated kinase (*P*-ERK; Takara Bio Inc., Shiga, Japan), total ERK (Abcam, Cambridge, UK), P65 and phospho-P65 (Abcam, Cambridge, UK), and phospho-P38 and total P38 (Abcam, Cambridge, UK). Complementary DNA synthesis was performed using a PrimerScript reverse transcriptase kit (Roche Diagnostics, Basel, Switzerland), following the manufacturer’s protocol.

#### 2.1.2. Chemical Inhibitors

Prostaglandin receptor antagonists and signaling pathway inhibitors were employed as follows: AH6809 (EP2 antagonist), and the compounds AH23848 (EP4 receptor blocker), CAY10404 (cyclooxygenase-2 suppressor), and MF63 (microsomal prostaglandin E synthase-1 inhibitor), which were sourced from Cayman Chemical Corporation (Ann Arbor, MI, USA). These pharmacological agents were used for experimental purposes.

#### 2.1.3. Equipment

Electrophoresis was performed using a power supply system (Beyotime Biotechnology, Shanghai, China).

### 2.2. Isolation, Culture, and Identification of Neutrophils in Dairy Cows

Blood samples were collected from healthy adult dairy cows (n = 3) via jugular vein puncture using sterile heparin sodium vacuum blood collection tubes. Each of the three collected blood samples was designated as an independent parallel group and experiments were performed concurrently. Bovine neutrophils were isolated using a commercial peripheral blood separation kit (Hao Yang Biotech, Singapore). The procedure was initiated by dispensing 4 mL of the primary separation medium into the centrifugation vessels. Fresh blood samples (3 mL) were carefully stratified over the separation medium along the tube walls, followed by 5 min centrifugation at 2000 rpm.

The intermediate leukocyte-rich phase (approximately 3 mL) was harvested and transferred to secondary tubes containing 2 mL of separation medium. Subsequent centrifugation proceeded at 2000 rpm for 40 min. After removing the supernatant, the cellular sediment was subjected to erythrocyte lysis.

A final centrifugation step (800× *g*, 8 min, and 23 °C) preceded cellular resuspension and quantification. The isolated neutrophils were plated onto culture dishes for subsequent analyses. Cellular characterization was performed by Wright-Giemsa differential staining to confirm neutrophil purity.

### 2.3. Molecular Docking

The gene name of the target protein was searched in the UniProt database to retrieve its UniProt identifier. Next, in the AlphaFold database, this UniProt identifier was input to download the three-dimensional structure of the corresponding target protein and save it as a PDB file. PyMOL software (Incentive Product, version 2.3.5; Schrödinger, LLC) was used to preprocess the downloaded protein structure by removing water and ligand molecules. The two-dimensional structure of the active ingredient was obtained from the PubChem database and saved as an SDF file, which was subsequently converted into a PDB file. AutoDock software (version 1.1.2) was utilized to preprocess the active ingredient, including defining rotatable bonds and specifying the docking center, and to export the processed file in the PDBQT format. Finally, the receptor (protein) and ligand (small molecule) files were imported into AutoDock software, a semi-flexible docking prediction analysis was performed, and the results were visualized using PyMOL software.

### 2.4. Effects of Various Inhibitors on Cytokine Production in Bovine Neutrophils Induced by S. aureus

Inoculate bovine neutrophils (n = 3) into a 6-well culture plate at a density of 2 × 10^6^ cells per well, and incubate the cells in a 37 °C, 5% CO_2_ cell incubator. Following cell adhesion to the plate surface, randomly assign them to the following treatment groups:

① Control group: The isolated and cultured bovine neutrophils were not subjected to bacterial stimulation or pharmacological treatment.

② *SA113* induction groups: Bovine neutrophils were induced with *SA113* at multiplicity of infection (MOI) ratios of 3:1 and 10:1, referred to as the *SA113* (MOI 3:1) group and the *SA113* (MOI 10:1) group, respectively.

③ *SA113* + CAY10404 treatment group: Following a 2 h pretreatment with CAY10404 (10^−5^ M), bovine neutrophils were stimulated with *SA113* at multiplicity of MOI ratios of 3:1 and 10:1, referred to as the *SA113* (MOI 3:1) + CAY10404 (10^−5^ M) and the *SA113* (MOI 10:1) + CAY10404 (10^−5^ M) group, respectively.

④ *SA113* + MF63 treatment group: Bovine neutrophils were pretreated with MF63 (10^−5^ M) for 24 h, followed by *SA113* induction at a MOI of 3:1 and 10:1. These groups were designated as the *SA113* (MOI 3:1) + MF63 (10^−5^ M) group and the *SA113* (MOI 10:1) + MF63 (10^−5^ M) group.

⑤ *SA113* + AH6809 treatment group: Bovine neutrophils were pretreated with AH6809 (10^−5^ M) for 1 h, followed by *SA113* induction at an MOI of 3:1 and 10:1. These groups were designated as the *SA113* (MOI 3:1) + AH6809 (10^−5^ M) group and the *SA113* (MOI 10:1) + AH6809 (10^−5^ M) group.

⑥ *SA113* + AH23848 treatment group: Bovine neutrophils were pretreated with AH23848 (10^−5^ M) for 1 h, followed by *SA113* induction at an MOI of 3:1 and 10:1. These groups were designated as the *SA113* (MOI 3:1) + AH23848 (10^−5^ M) group and the *SA113* (MOI 10:1) + AH23848 (10^−5^ M) group.

1 h after bacterial induction, gentamicin sulfate was added to each treatment group at a final concentration of 100 µg/mL to eliminate extracellular *S. aureus* and prevent excessive bacterial proliferation that could lead to host cell death. At 9 h after treatment, cell supernatants were collected and stored at −80 °C until further analysis.

Subsequently, according to the specific protocols outlined in the respective ELISA assay kits, calibration curves were generated as required. The production quantities of multiple inflammatory mediators (IL-1β, IL-6, and IL-10), as well as PGE_2_, were separately quantified. The cytokine concentrations in the samples were calculated using established calibration references. To guarantee measurement accuracy, biological repetitions were performed in triplicate for each test specimen.

### 2.5. Effects of Various Inhibitors on the Activation of MAPK and NF-κB Signaling Pathways in Bovine Neutrophils Induced by S. aureus

Inoculate bovine neutrophils (n = 3) into a 6-well culture plate at a density of 2 × 10^6^ cells per well, and incubate the cells in a 37 °C, 5% CO_2_ cell incubator. Following cell adhesion to the plate surface, randomly assign them to the following treatment groups:

① Blank control group: The isolated and cultured dairy cow neutrophils were not exposed to any bacterial or pharmacological stimulation.

② *SA113* induction group: Dairy cow neutrophils were stimulated with *SA113* at a multiplicity of infection (MOI) of 10:1.

③ Inhibitor treatment groups: CAY10404, MF63, AH6809, and AH23848 were applied to dairy cow neutrophils at a concentration of 10^−5^ M and allowed to act for 2 h, 24 h, 1 h, and 1 h, respectively.

④ *SA113* + CAY10404 treatment group: Dairy cow neutrophils were first pretreated with 10^−5^ M CAY10404 for 2 h, followed by induction with *SA113* at an MOI of 10:1 for 15 min, 30 min, and 60 min, respectively.

⑤ *SA113* + MF63 treatment group: Dairy cow neutrophils were initially pretreated with 10^−5^ M MF63 for 24 h, then induced with *SA113* at an MOI of 10:1 for 15 min, 30 min, and 60 min, respectively.

⑥ *SA113* + AH6809 treatment group: Dairy cow neutrophils were pretreated with 10^−5^ M AH6809 for 1 h, followed by induction with *SA113* at an MOI of 10:1 for 15 min, 30 min, and 60 min, respectively.

⑦ *SA113* + AH23848 treatment group: Dairy cow neutrophils were pretreated with 10^−5^ M AH23848 for 1 h, followed by induction with *SA113* at an MOI of 10:1 for 15 min, 30 min, and 60 min, respectively.

After bacterial induction was completed, gentamicin sulfate was added to a final concentration of 100 µg/mL in order to eliminate extracellular *S. aureus*. Protein extraction was performed by cell lysis with M-PER solution, with subsequent isolation of total proteins conducted under chilled conditions prior to concentration assessment and denaturation. For electrophoresis analysis, 20 µg of protein specimens underwent electrophoretic fractionation using 12% sodium dodecyl sulfate-polyacrylamide gel matrices. The separation process employed discontinuous buffer systems under denaturing conditions, with molecular weight resolution achieved through controlled pore size gradients.

Electrophoretic migration occurred at constant voltage until the tracking dye reached the distal boundary of the gel, ensuring complete polypeptide chain separation based on hydrodynamic radii, and was then electro transferred onto PVDF membranes. Membrane blocking was achieved by incubation with 3% bovine serum albumin in TBST solution for 4 h at ambient temperature. Primary antibodies at optimal dilutions were applied for overnight binding at 4 °C overnight. After three TBST washing cycles, the secondary antibody was incubated for 60 min at room temperature. The post-wash development employed ECL detection with quantitative band analysis using ImageJ software (version 1.50b). All the experiments were performed in triplicate.

### 2.6. Effect of Various Inhibitors on Phagocytic Activity of Dairy Cow Neutrophils

① Bacterial Staining: Stain live *SA113* cells with Hoechst dye. Following a 0.5 h staining period in the dark, wash the cells three times with PBS and reserve them for subsequent procedures.

② Cell Membrane Staining: Add CAY10404, MF63, AH6809, and AH23848 to cow neutrophils (n = 3) at a final concentration of 10^−5^ M, and allow each compound to act for 2 h, 24 h, 1 h, and 1 h, respectively. After treatment, introduce DiI dye and incubate the samples in the dark for 30 min.

③ Co-incubation of Bacteria and Cells: Inoculate the stained *SA113* bacteria into the stained neutrophils at a multiplicity of infection (MOI) of 30:1 and incubate at 37 °C for 30 min. Subsequently, fix the cells using formaldehyde. Finally, images of the fixed cells were captured using a laser confocal microscope (LSM 800; Carl Zeiss AG, Jena, Germany), and the fluorescence intensity was quantitatively analyzed with Zen software (Blue, Carl Zeiss AG, Germany). All the experiments were performed in triplicate.

### 2.7. Statistical Analysis

All the data were analyzed using Prism software (GraphPad Software, Prism 10.0, Boston, MA, USA). For measurement data, normality was assessed via the Shapiro–Wilk test and homogeneity of variance was tested to determine whether parametric assumptions were satisfied. Data meeting normality and homogeneity criteria were presented as mean ± standard deviation ($\bar{x}$ ± SD). For comparisons among multiple treatment groups, one-way analysis of variance (ANOVA) was employed. If the overall difference was statistically significant (*p* < 0.05), post hoc pairwise comparisons were conducted using the Tukey method. To evaluate the interaction effects between different treatment groups and time points, two-way ANOVA was applied, followed by the Tukey method for multiple comparisons. Significant differences between the experimental group and the control group were indicated with the #: ns for *p* > 0.05 (no significant difference), # for *p* < 0.05, ## for *p* < 0.01, and ### for *p* < 0.001. Comparisons showing significant differences between two designated groups were marked with the *: ns for *p* > 0.05, * for *p* < 0.05, ** for *p* < 0.01, and *** for *p* < 0.001.

## 3. Results

### 3.1. Effects of COX-2 and mPGES-1 Inhibitors on Expression of Inflammatory Factors in Dairy Cow Neutrophils Induced by S. aureus

#### 3.1.1. Verification of Molecular Docking for Inhibitors

In this study, two inhibitors, CAY10404 and MF63, were utilized to target and suppress COX-2 (AlphaFoldDB: AF-O62698-F1) and mPGES-1 (AlphaFoldDB: AF-Q95L14-F1), which are crucial catalytic elements within the PGE_2_ production mechanism observed in bovine neutrophils. The intervention focused on the enzymatic components known to mediate inflammatory responses in lactating cows. This was carried out to investigate whether PGE_2_ influences secretion and expression of inflammatory factors in *SA113*-induced dairy cow neutrophils. Preliminary computational analysis employed molecular docking techniques to verify specific interactions between the catalytic domains of CAY10404 and COX-2, while simultaneously assessing the binding preferences of MF63 toward the active regions of mPGES-1.

**Figure 1:**
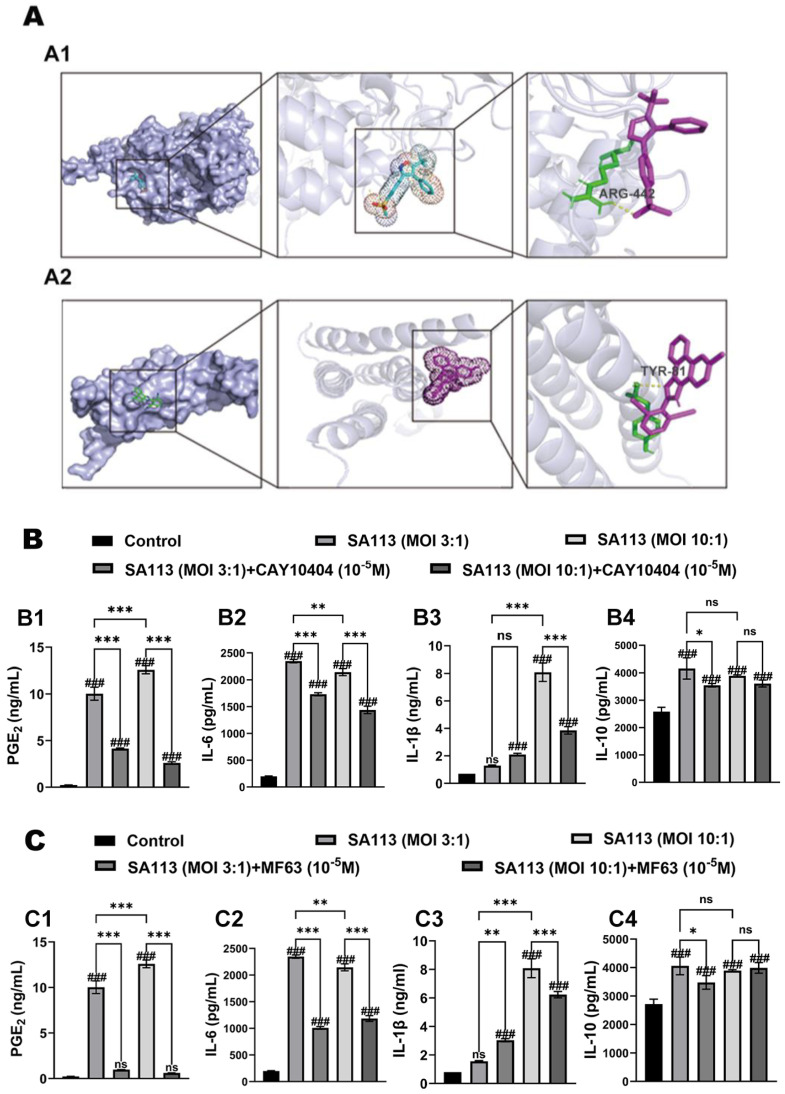
Effect of COX-2 inhibitor and mPGES-1 inhibitor on PGE_2_ production and inflammatory mediator secretion in bovine neutrophil challenged with *S. aureus*. (**A**) Molecular docking results of two inhibitors: (**A1**) COX-2 (AlphaFoldDB: AF-O62698-F1) Inhibitor (CAY10404) and (**A2**) mPGES-1 (AlphaFoldDB: AF-Q95L14-F1) Inhibitor (MF63). The effects of pretreatment with the (**B**) COX-2 inhibitor (CAY10404) and the (**C**) mPGES-1 inhibitor (MF63) on the expression levels of (**B1**,**C1**) PGE_2_ and inflammatory factors including (**B2**,**C2**) IL-6, (**B3**,**C3**) IL-1β, and (**B4**,**C4**) IL-10 in *S. aureus*-induced bovine neutrophils. Results are expressed as mean ± standard deviation of three independent experiments. To compare differences among multiple treatment groups, one-way ANOVA was employed. If the overall difference was statistically significant (*p* < 0.05), post hoc pairwise comparisons were conducted using the Tukey method. Significant differences between the experimental group and the control group were indicated with the #: ns for *p* > 0.05 (no significant difference), and ### for *p* < 0.001. Comparisons showing significant differences between two designated groups were marked with the *: ns for *p* > 0.05, * for *p* < 0.05, ** for *p* < 0.01, and *** for *p* < 0.001. Abbreviations: MOI, multiplicity of infection; ARG-442, arginine at position 442; TYR-81, tyrosine at position 81; COX-2, Cyclooxygenase-2; mPGES-1, Microsomal prostaglandin E synthase-1; PGE_2_, Prostaglandin E_2_; IL-6, Interleukin-6; IL-1β, Interleukin-1 beta; IL-10, Interleukin-10.

The molecular docking results are shown in Figure 1. As shown in Figure 1(A1), CAY10440 docked with COX-2 at its active center, forming hydrogen bonds with arginine at position 442 of COX-2. As shown in Figure 1(A2), MF63 docked with mPGES-1, also at the active center of the enzyme, forming hydrogen bonds with tyrosine at position 81 of mPGES-1. The experimental results indicated that CAY10404 demonstrated dual inhibitory effects on COX-2 and mPGES-1 enzymatic activity, mirroring the pharmacological profile of MF63 when administered to dairy cows.

#### 3.1.2. Effect of COX-2 and mPGES-1 Inhibitors on Expression Levels of PGE_2_ and Inflammatory Factors in Dairy Cow Neutrophils Induced by *S. aureus* Infection

In this study, neutrophils isolated from dairy cows were pretreated with the COX-2 inhibitor CAY10404 and mPGES-1 inhibitor MF63, and were then stimulated with *SA113* to induce an inflammatory response. Endogenous PGE_2_ levels were quantified using ELISA. As shown in Figure 1(B1,C1), following treatment with both inhibitors (CAY10404 and MF63), the PGE_2_ production and release from neutrophils stimulated with *S. aureus* showed a marked decrease compared to *SA113*-triggered samples (*p* < 0.001).

Subsequently, IL-6, IL-1β, and IL-10 expression levels were measured using ELISA to investigate whether endogenous PGE_2_ was involved in the inflammation induced by *SA113*-stimulated bovine neutrophils. As shown in Figure 1B, C, IL-6, IL-1β, and IL-10 concentrations exhibited marked elevation upon stimulation of bovine neutrophils with *SA113* (*p* < 0.05).

Pretreatment with the COX-2 inhibitor CAY10404 prior to *SA113* induction significantly suppressed the secretion of IL-6 (Figure 1(B2)). Compared to the *SA113*-exposed cohort, the addition of CAY10404 intervention markedly suppressed IL-6 production (*p* < 0.001). Figure 1(B3) presents comparative IL-1β expression data, revealing no statistically meaningful variation between *SA113*-stimulated and CAY10404-pretreated bovine neutrophils at lower infection ratios (3:1) (*p* > 0.05). Elevated pathogen exposure (10:1 ratio) triggered substantial IL-1β overexpression in *SA113*-challenged specimens versus that in untreated controls (*p* < 0.001), whereas COX-2 inhibition dramatically attenuated this response (*p* < 0.001).

The immunomodulatory cytokine IL-10 exhibited different regulatory patterns (Figure 1(B4)). Under limited bacterial challenge conditions (3:1 MOI), CAY10404 preconditioning caused statistically detectable IL-10 suppression relative to *SA113* monotherapy (*p* < 0.05); however, this inhibitory effect was not significant (*p* > 0.05) when the infection intensity was increased to 10:1 MOI.

Following pretreatment with MF63 (mPGES-1 inhibitor), cytokine secretion patterns across the experimental groups were quantitatively assessed (Figure 1C). Pharmacological interventions significantly modulated the release profiles of multiple inflammatory mediators. The trends in cytokine expression levels were largely consistent with those observed following pretreatment with the COX-2 inhibitor, CAY10404. Specifically, at MOI ratios of 3:1 and 10:1, the selective mPGES-1 inhibitor MF63 markedly reduced the *SA113* infection-triggered elevation in IL-6 production (*p* < 0.001) (Figure 1(C2)). This pharmacological intervention effectively counteracted pathogen-induced cytokine upregulation. In contrast, its inhibitory effect on IL-1β expression was more pronounced at an MOI of 10:1 (*p* < 0.001) (Figure 1(C3)). Additionally, suppression of IL-10 expression was relatively weak and was significant only at an MOI of 3:1 (*p* < 0.05) (Figure 1(C4)).

#### 3.1.3. Effects of COX-2 and mPGES-1 Inhibitors on Activation of MAPK and NF-κB Signaling Pathways in Dairy Cow Neutrophils Induced by *S. aureus* Infection

**Figure 2:**
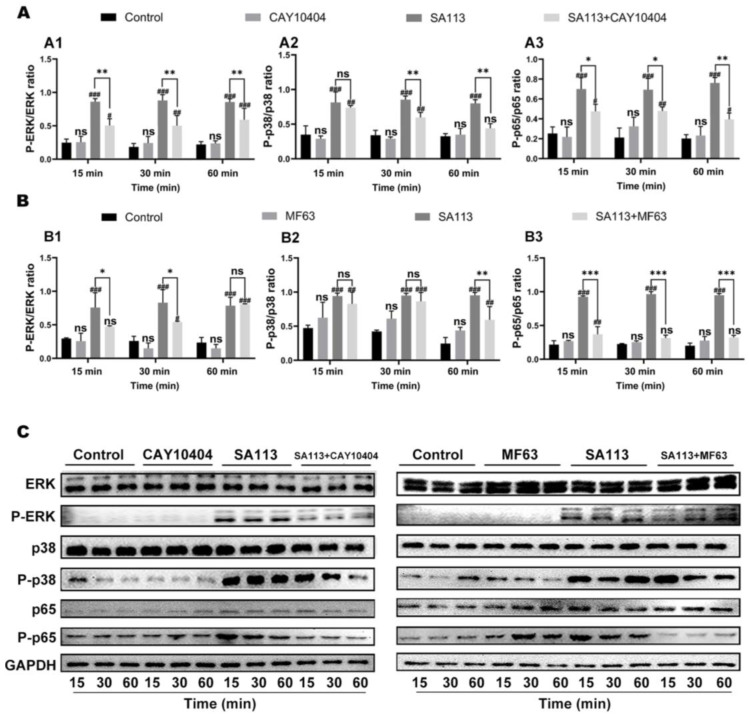
Effects of COX-2 and mPGES-1 inhibitors on MAPK/NF-κB pathway activation in bovine neutrophils during *S. aureus* infection. Bovine neutrophils were pretreated with either (**A**) the selective COX-2 inhibitor CAY10404 or (**B**) the mPGES-1-specific inhibitor MF63 prior to *S. aureus* exposure. Protein phosphorylation status was evaluated through immunoblotting for: (**A1**,**B1**) Extracellular signal-regulated kinase (ERK), (**A2**,**B2**) P38 MAP kinase, and (**A3**,**B3**) NF-κB p65 subunit. (**C**) Summary graph of WB bands. Results are expressed as mean ± standard deviation of three independent experiments. To evaluate the interaction effects between different treatment groups and time points, two-way ANOVA was applied, followed by the Tukey method for multiple comparisons. Significant differences between the experimental group and the control group were indicated with the #: ns for *p* > 0.05 (no significant difference), # for *p* < 0.05, ## for *p* < 0.01, and ### for *p* < 0.001. Comparisons showing significant differences between two designated groups were marked with the *: ns for *p* > 0.05, * for *p* < 0.05, ** for *p* < 0.01, and *** for *p* < 0.001. Abbreviations: MAPK, Mitogen-Activated Protein Kinase; NF-κB, Nuclear Factor kappa-light-chain-enhancer of activated B cells; COX-2, Cyclooxygenase-2; mPGES-1, Microsomal prostaglandin E synthase-1; ERK, Extracellular signal-regulated kinase; P-ERK, Phosphorylated extracellular signal-regulated kinase; p38, p38 mitogen-activated protein kinase; P-p38, Phosphorylated p38 mitogen-activated protein kinase; p65, Nuclear factor kappa-B subunit p65; P-p65, Phosphorylated nuclear factor kappa-B subunit p65; GAPDH, Glyceraldehyde-3-phosphate dehydrogenase. Original figures can be found in Supplementary Materials.

This study employed CAY10404 and MF63 as pretreatment agents to block activity of COX-2 and mPGES-1 (i.e., crucial enzymes involved in PGE_2_ production) in bovine neutrophils. Following this, *SA113* stimulation was administered, with subsequent evaluation of ERK, P38, and P65 phosphorylation states using Western blot analysis. Figure 2 demonstrates that relative to untreated controls, *SA113*-exposed neutrophils exhibited substantial elevation in phosphorylated ERK (Figure 2(A1)), P38 (Figure 2(A2)), and P65 (Figure 2(A3)) at 15-, 30-, and 60-min intervals, respectively (*p* < 0.001).

When COX-2 inhibition was implemented using CAY10404 prior to *SA113* exposure, there was pronounced attenuation of ERK and P65 phosphorylation across all time points relative to that under *SA113* stimulation alone (*p* < 0.05) (Figure 2(A1,A3)). Additionally, Figure 2(A2) indicates that the CAY10404-mediated suppression of P38 phosphorylation was statistically significant at 30 and 60 min (*p* < 0.01), although no significant inhibition was observed at 15 min (*p* > 0.05).

After pretreating the cells with the mPGES-1 inhibitor MF63, ERK phosphorylation in dairy cow neutrophils was significantly inhibited in the *SA113* + MF63 treatment group compared to that in the *SA113*-induced group at both 15 and 30 min (*p* < 0.05) (Figure 2(B1)). Figure 2(B2) indicates that P38 activation in dairy cow neutrophils was significantly suppressed in the *SA113* + MF63 treatment group compared to that in the *SA113*-induced group at 60 min (*p* < 0.01). Furthermore, Figure 2(B3) demonstrates that P65 activation in dairy cow neutrophils was significantly inhibited in the *SA113* + MF63 treatment group compared to that in the *SA113*-induced group at 15, 30, and 60 min (*p* < 0.001), respectively.

It can be inferred that upon adding the two inhibitors (CAY10404 and MF63) to dairy cow neutrophils induced by *S. aureus* to selectively inhibit the two key enzymes (COX-2 and mPGES-1) in the PGE_2_ synthesis pathway, activation of ERK, P38, and P65 was also suppressed. This suggested that PGE_2_ modulated secretion of inflammatory cytokines IL-6, IL-1β, and IL-10 via the MAPK and NF-κB signaling pathways.

### 3.2. Effects of EP2 and EP4 Receptor Inhibitors on the Post-Inflammatory Response of Dairy Cow Neutrophils Induced by S. aureus Infection

#### 3.2.1. Verification of Molecular Docking for Inhibitors

In this study, AH6809 and AH23848 were used to inhibit the EP2 (AlphaFoldDB: AF-Q8MJ09-F1) and EP4 (AlphaFoldDB: AF-Q8MJ08-F1) receptors of dairy cow neutrophils to verify whether endogenous PGE_2_ participates in *SA113*-induced secretion of related inflammatory factors in dairy cow neutrophils via the EP2/EP4 receptor pathway.

**Figure 3:**
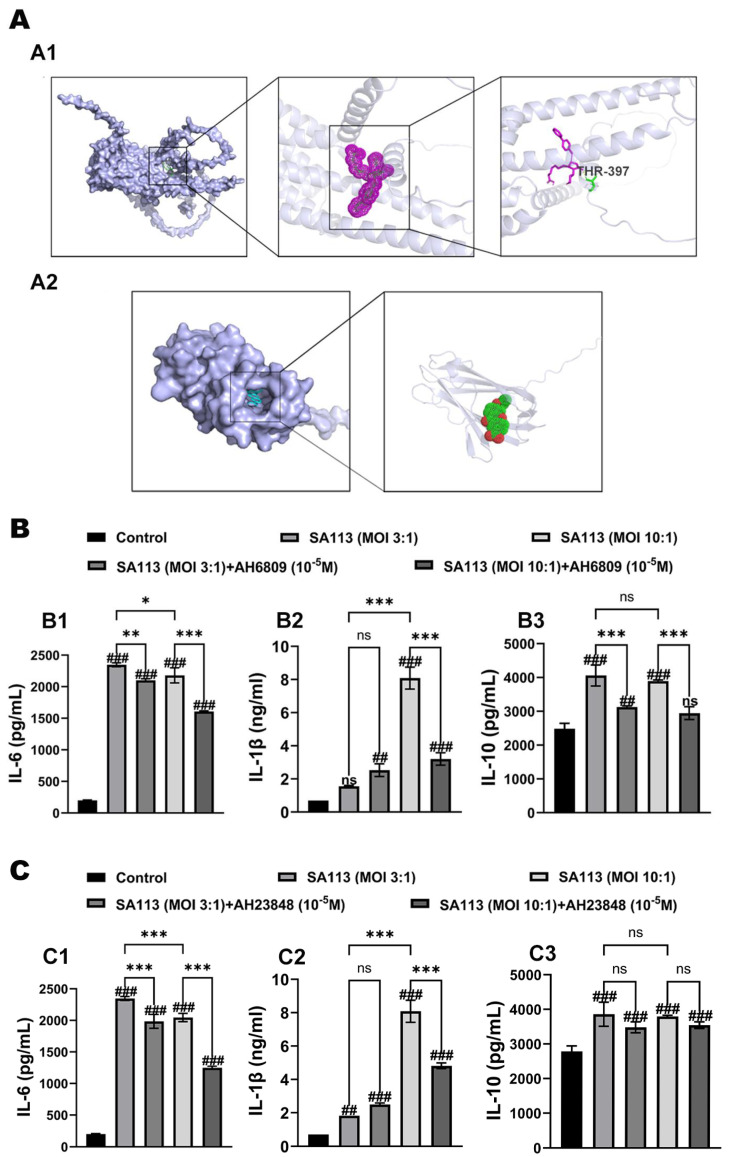
Effects of EP2 and EP4 receptor inhibitors on expression levels of inflammatory factors in dairy cow neutrophils induced by *S. aureus* infection. (**A**) Molecular docking results of two inhibitors: (**A1**) EP4 (AlphaFoldDB: AF-Q8MJ08-F1) receptor inhibitor (AH23848) and (**A2**) EP2 (AlphaFoldDB: AF-Q8MJ09-F1) receptor inhibitor (AH6809).The effects of pretreatment with the (**B**) EP2 receptor inhibitor AH6809 and (**C**) the EP4 receptor inhibitor AH23848 on the expression levels of inflammatory cytokines in *S. aureus*-induced bovine neutrophils: (**B1**,**C1**) IL-6, (**B2**,**C2**) IL-1β, and (**B3**,**C3**) IL-10. Results are expressed as mean ± standard deviation of three independent experiments. To compare differences among multiple treatment groups, one-way ANOVA was employed. If the overall difference was statistically significant (*p* < 0.05), post hoc pairwise comparisons were conducted using the Tukey method. Significant differences between the experimental group and the control group were indicated with the #: ns for *p* > 0.05 (no significant difference), ## for *p* < 0.01, and ### for *p* < 0.001. Comparisons showing significant differences between two designated groups were marked with the *: ns for *p* > 0.05, * for *p* < 0.05, ** for *p* < 0.01, and *** for *p* < 0.001. Abbreviations: EP2 receptor, Prostaglandin E_2_ receptor EP2 subtype; EP4 receptor, Prostaglandin E_2_ receptor EP4 subtype; MOI, multiplicity of infection; THR-397, threonine at position 397; IL-6, Interleukin-1 beta; IL-10, Interleukin-10.

Initially, the binding sites of the two receptor antagonists and their corresponding receptors were predicted by molecular docking. The molecular docking results are shown in Figure 3A. As shown in Figure 3(A1), the docking results revealed that AH23848 bound to the active center of the EP4 receptor through hydrogen bonds with threonine at position 397. Similarly, as depicted in Figure 3(A2), AH6809 docked to the active center of the EP2 receptor. The results demonstrated that both substances successfully interacted with bovine EP2 and EP4 receptor proteins.

#### 3.2.2. Effects of EP2 and EP4 Receptor Inhibitors on Cytokine Expression in Dairy Cow Neutrophils Induced by *S. aureus* Infection

In this study, dairy cow neutrophils were pretreated with two receptor inhibitors, AH6809 and AH23848, and *SA113* was introduced for induction. The alterations in IL-6, IL-1β, and IL-10 expression levels were quantified using ELISA methodology, thereby verifying whether PGE_2_ participates in the *SA113*-induced secretion of related inflammatory factors in dairy cow neutrophils via the EP2/EP4 receptor pathway.

When pretreated with the EP2 receptor inhibitor AH6809, the secretion levels of various cytokines in each group are presented in Figure 3B. As shown in Figure 3(B1), compared to the blank control group, the expression of IL-6 in dairy cow neutrophils in the *SA113*-induced group was significantly higher (*p* < 0.001). Pretreatment with the EP2 receptor inhibitor AH6809 markedly attenuated this increase (*p* < 0.01). As depicted in Figure 3(B2), at an MOI of 10:1, the expression of IL-1β in dairy cow neutrophils in the *SA113*-induced group was significantly higher than that in the blank control group (*p* < 0.001). Furthermore, pretreatment with the EP2 receptor inhibitor AH6809 led to a significant reduction in IL-1β expression compared to the *SA113*-induced group (*p* < 0.001). As illustrated in Figure 3(B3), compared to the blank control group, IL-10 expression in dairy cow neutrophils was significantly upregulated following *SA113* induction (*p* < 0.001). This upregulation was significantly mitigated by pretreatment with the EP2 receptor inhibitor AH6809 (*p* < 0.001).

After pretreatment with the EP4 receptor inhibitor AH23848, the secretion levels of various cytokines in each group are presented in Figure 3C. As shown in Figure 3(C1), in contrast to the *SA113* induction cohort, neutrophil IL-6 production was markedly inhibited in the *SA113* + AH23848 intervention arm (*p* < 0.001). Figure 3(C2) demonstrates that at an MOI of 10:1, IL-1β expression in dairy cow neutrophils was also markedly inhibited in the *SA113* + AH23848 group relative to that in the *SA113* induction group (*p* < 0.001). Figure 3(C3) shows that at all infection ratios, although neutrophil IL-10 levels exhibited a marginal reduction in the *SA113* + AH23848 cohort relative to the *SA113*-stimulated animals, this variation was not statistically significant (*p* > 0.05).

In summary, following the addition of the two receptor inhibitors, AH6809 and AH23848, to the neutrophils of dairy cows and subsequent induction with *SA113*, a notable reduction was detected in the release levels of inflammatory mediators, including IL-6, IL-1β, and IL-10, across different experimental conditions. The collected data imply that PGE2 participates in modulating neutrophil-derived cytokine production in bovine specimens following *SA113* stimulation, primarily through signaling mechanisms involving the EP2 and EP4 receptor subtypes.

#### 3.2.3. Effects of EP2 and EP4 Receptor Inhibition on Activation of MAPK and NF-κB Pathways in Dairy Cow Neutrophils Induced by *S. aureus* Infection

During this experimental procedure, neutrophils obtained from bovine dairy cows were treated with EP2 and EP4 receptor antagonists prior to stimulation with *SA113*. Subsequent analysis through Western blotting revealed the phosphorylation status of ERK, P38, and P65 critical components within both the MAPK cascade and NF-κB signaling network.

**Figure 4:**
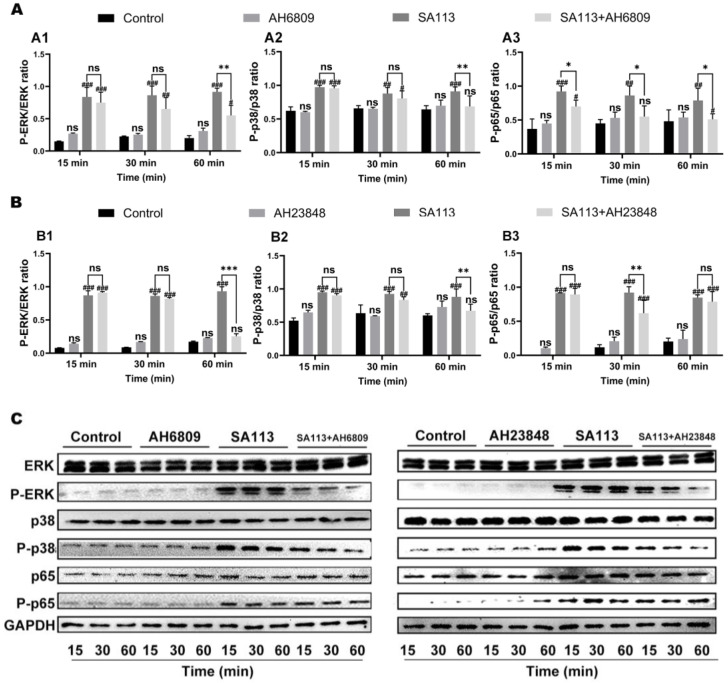
Influence of EP2 and EP4 receptor antagonists on MAPK and NF-κB pathway stimulation in bovine neutrophils exposed to *S. aureus*. Bovine neutrophils were pretreated with either (**A**) the EP2 receptor antagonist AH6809 or (**B**) the EP4 receptor blocker AH23848. Protein phosphorylation status was evaluated through immunoblotting for: (**A1**,**B1**) Extracellular signal-regulated kinase (ERK), (**A2**,**B2**) P38 MAP kinase, and (**A3**,**B3**) NF-κB p65 subunit. (**C**) Summary graph of WB bands. Results are expressed as mean ± standard deviation of three independent experiments. To evaluate the interaction effects between different treatment groups and time points, two-way ANOVA was applied, followed by the Tukey method for multiple comparisons. Significant differences between the experimental group and the control group were indicated with the #: ns for *p* > 0.05 (no significant difference), # for *p* < 0.05, ## for *p* < 0.01, and ### for *p* < 0.001. Comparisons showing significant differences between two designated groups were marked with the *: ns for *p* > 0.05, * for *p* < 0.05, ** for *p* < 0.01, and *** for *p* < 0.001. Abbreviations: MAPK, Mitogen-Activated Protein Kinase; NF-κB, Nuclear Factor kappa-light-chain-enhancer of activated B cells; EP2 receptor, Prostaglandin E_2_ receptor EP2 subtype; EP4 receptor, Prostaglandin E_2_ receptor EP4 subtype; ERK, Extracellular signal-regulated kinase; P-ERK, Phosphorylated extracellular signal-regulated kinase; p38, p38 mitogen-activated protein kinase; P-p38, Phosphorylated p38 mitogen-activated protein kinase; p65, Nuclear factor kappa-B subunit p65; P-p65, Phosphorylated nuclear factor kappa-B subunit p65; GAPDH, Glyceraldehyde-3-phosphate dehydrogenase. Original figures can be found in Supplementary Materials.

As shown in Figure 4, compared to the blank control group, activation of ERK, P38, and P65 in dairy cow neutrophils in the *SA113*-induced group was significantly upregulated at 15, 30, and 60 min (*p* < 0.01). As shown in Figure 4(A1,A2), compared with the *SA113*-induced group, there was no significant difference in ERK and P38 activation in dairy cow neutrophils in the *SA113* + AH6809 treatment group at 15 or 30 min (*p* > 0.05); however, activation was significantly reduced at 60 min (*p* < 0.01). As shown in Figure 4(A3), at each treatment time point, the phosphorylation level of P65 in dairy cow neutrophils in the *SA113* + AH6809 treatment group was significantly lower than that in the *SA113*-induced group (*p* < 0.05).

As shown in Figure 4(B1), compared to the *SA113* induction group, there was no significant difference in ERK activation in dairy cow neutrophils in the *SA113* + AH23848 treatment group at 15 or 30 min (*p* > 0.05); however, activation was significantly inhibited at 60 min (*p* < 0.001). As depicted in Figure 4(B2), compared to the *SA113* induction group, P38 activation in dairy cow neutrophils in the *SA113* + AH23848 treatment group was significantly suppressed at 60 min (*p* < 0.01). Figure 4(B3) shows that, compared with the *SA113* induction group, P65 activation in dairy cow neutrophils in the *SA113* + AH23848 treatment group was significantly downregulated at 30 min (*p* < 0.01), whereas no significant differences were noted at 15 or 60 min (*p* > 0.05).

It can be inferred from these results that after adding EP2 and EP4 receptor inhibitors to neutrophils of dairy cows and subsequently inducing them with *SA113*, the phosphorylation levels of ERK, P38, and P65—key proteins in the MAPK and NF-κB signaling pathways—were downregulated to varying extents. Based on these experimental findings, it is reasonable to conclude that PGE_2_ mediated the activation of the NF-κB and MAPK inflammatory signaling pathways via the EP2/EP4 receptor pathway, thereby modulating the expression and secretion of pro- inflammatory and anti-inflammatory cytokines.

### 3.3. Effect of COX-2 and mPGES-1 Inhibitors, as Well as EP2 and EP4 Receptor Antagonists, on the Phagocytic Activity of Neutrophils in Dairy Cows Against S. aureus Infection

#### 3.3.1. Effect of COX-2 and mPGES-1 Inhibitors on Phagocytic Activity of Neutrophils in Dairy Cows Against *S. aureus* Infection

In this study, two inhibitors (CAY10404 and MF63) were added to the neutrophils of dairy cows to inhibit activities of COX-2 and mPGES-1, respectively, which are key enzymes in the PGE_2_ synthesis pathway. Subsequently, *SA113* bacteria labeled with Hoechst dye were co-incubated with the treated neutrophils. Phagocytic activity was evaluated by measuring the average fluorescence intensity to assess the impact of COX-2 and mPGES-1 inhibition on neutrophils phagocytic function.

**Figure 5:**
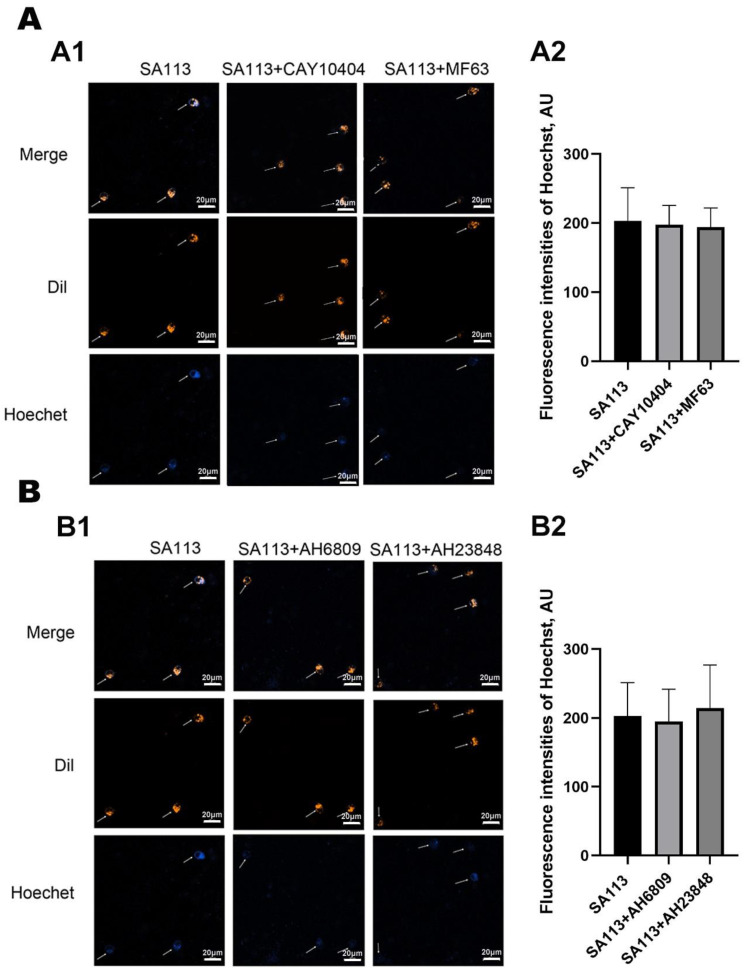
Effect of COX-2 and mPGES-1 inhibitors, as well as EP2 and EP4 inhibitors, on the phagocytic activity of neutrophils in dairy cows against *S. aureus* Infection. (**A**) Effects of the COX-2 inhibitor CAY10404 and the mPGES-1 inhibitor MF63 on phagocytic performance in bovine neutrophils activated by *S. aureus* exposure. (**A1**) Laser confocal microscopic images. In these images, the DiI dye was used to label the cell membrane, while the Hoechst dye was applied to stain *SA113*. (**A2**) The average fluorescence intensity of Hoechst dye in each treatment group. (**B**) Effects of the EP2 receptor antagonist AH6809 and the EP4 receptor antagonist AH23848 on the phagocytic functionality of bovine neutrophils exposed to *S. aureus*. (**B1**) Laser confocal microscopic images. In these images, the DiI dye was used to label the cell membrane, while the Hoechst dye was applied to stain *SA113*. (**B2**) The average fluorescence intensity of Hoechst dye in each treatment group. Scale: 20 µm. Results are expressed as mean ± standard deviation of three independent experiments. To compare differences among multiple treatment groups, one-way ANOVA was employed. If the overall difference was statistically significant (*p* < 0.05), post hoc pairwise comparisons were conducted using the Tukey method. COX-2, Cyclooxygenase-2; mPGES-1, Microsomal prostaglandin E synthase-1; EP2 receptor, Prostaglandin E_2_ receptor EP2 subtype; EP4 receptor, Prostaglandin E_2_ receptor EP4 subtype; DiI, 1,1′-Dioctadecyl-3,3,3′,3′-Tetramethylindocarbocyanine Perchlorate.

The data presented in Figure 5A demonstrated that neutrophil fluorescence intensity remained statistically unchanged when exposed to COX-2 and mPGES-1 inhibitors relative to that in untreated controls (*p* > 0.05). These findings indicated that blocking COX-2 and mPGES-1 enzymatic activity failed to alter neutrophil phagocytic capacity under the experimental conditions.

#### 3.3.2. Effect of EP2 and EP4 Receptor Antagonists on Phagocytic Activity of Neutrophils in Dairy Cows Against *S. aureus* Infection

In this study, EP2/EP4 receptors in dairy cow neutrophils were inhibited using two specific inhibitors, AH6809 and AH23848. Subsequently, cells were treated with *SA113*. Immunofluorescence analysis was used to evaluate the effect of blocking EP2/EP4 receptors on the phagocytic activity of bovine neutrophils. Figure 5B shows comparable fluorescence intensity levels across the *SA113*-exposed and intervention groups (*SA113* combined with AH6809 or AH23848) (*p* > 0.05). Consequently, these findings suggested that suppression of EP2/EP4 receptor signaling failed to alter neutrophil phagocytic capacity in lactating cows.

## 4. Discussion

*S. aureus* can induce a range of postpartum diseases, including mastitis and vaginitis, in dairy cows, leading to substantial culling rates and reduced milk production [34,35,36]. Following *S. aureus* infection, the cellular components of the immune system become activated, generating substantial quantities of pro-inflammatory mediators, including IL-1β, IL-6, and IL-10, which are crucial for mounting protective responses [37]. However, excessive cytokine production may result in multiple organ dysfunction syndromes and even mortality. Previous research from our laboratory demonstrated that *S. aureus* induces an inflammatory response in dairy cow neutrophils via natural immune receptors (TLR2, TLR4, and NLRP3), which also participate in and regulate PGE_2_ synthesis and secretion in neutrophils [38,39]. Research findings demonstrate that PGE_2_ predominantly regulates inflammatory reactions caused by bacterial infections by binding to EP2 and EP4 receptor subtypes [40]. Nevertheless, the specific mechanisms by which PGE_2_ functions in *S. aureus*-induced dairy cow neutrophils remain unclear. Therefore, this study investigated the regulatory role of the endogenous PGE_2_-EP2/4 receptor pathway in the inflammatory response induced by *S. aureus* in dairy cow neutrophils, and elucidated its underlying mechanism. This study provides a theoretical foundation for the prevention of bovine diseases associated with *S. aureus* in clinical veterinary practice, and establishes a scientific basis for identifying potential targets in the development of novel therapeutic agents. Additionally, this study provides empirical evidence supporting the judicious use of non-steroidal anti-inflammatory medications for managing bacterial infection-related inflammatory conditions in animal healthcare.

Because the effectiveness of the inhibitors selected in this study has not been verified in dairy cows, and particularly because mPGES-1 inhibitors exhibit strong species specificity and are not broadly applicable to most animals [24,25,26], it was necessary to evaluate their potential efficacy. Therefore, in this study, a computational docking analysis was performed to examine the interactions between COX-2/mPGES-1 inhibitors (CAY10404 and MF63) and EP2/EP4 receptor antagonists (AH6809 and AH23848). These results indicated that the more stable the conformation of the receptor–ligand complex, the lower its binding energy and the greater the effect produced, suggesting a high affinity between the main active components and their targets [41]. The investigation revealed that all tested compounds formed stable complexes within receptor-binding pockets, characterized by favorable energy profiles, indicating strong binding activity between the drugs and their corresponding targets.

Subsequently, in this study, *S. aureus* was used to activate neutrophils isolated from bovine bone marrow, with subsequent measurement of the inflammatory mediator concentrations. Statistical analysis demonstrated significant upregulation of inflammatory mediators (IL-1β and IL-6) alongside the regulatory cytokine IL-10 after microbial challenge. Concurrent measurements revealed a proportional increase in PGE_2_ synthesis, implying its potential involvement in modulating cytokine release during *Staphylococcal* infections of bovine neutrophils. These observations are consistent with prior research conducted by Gill et al. (2016) demonstrating PGE_2_-mediated suppression of inflammatory responses in human pulmonary macrophages through EP4 receptor signaling [42]. Subsequent experimental manipulation employed the COX-2 inhibitor, CAY10404, and the mPGES-1 inhibitor, MF63, to suppress PGE_2_ biosynthesis, thereby examining its functional relationship with cytokine production. Inhibition of PGE_2_ synthesis resulted in a corresponding reduction in both pro- and anti-inflammatory cytokine secretion, confirming its regulatory influence on *Staphylococcus*-induced immune responses. Th existing literature documents the critical involvement of NF-κB and MAPK pathways in *S. aureus*-triggered inflammatory mediator production [43]. Accordingly, this study examined the effects of COX-2 and mPGES-1 inhibition on the activation of signaling pathways in pathogen-exposed bovine neutrophils. The experimental data demonstrated that pharmacological blockade of these enzymes differentially attenuated ERK, P38, and P65 phosphorylation. Therefore, during the process of *S. aureus*-induced bovine neutrophil activation, COX-2 and mPGES-1 played crucial roles in PGE_2_ production and release. This biologically active lipid compound modulated the secretion of pro- and anti-inflammatory cytokines by engaging with the NF-κB and MAPK signal transduction pathways, and molecular interactions altered cytokine production through complex regulatory mechanisms involving key signaling networks.

To investigate whether the PGE_2_-EP2/EP4 receptor pathway regulated secretion of inflammatory factors in dairy cow neutrophils induced by *S. aureus*, we analyzed the way in which AH6809 and AH23848, acting as EP2/EP4 receptor blockers, influenced associated inflammatory processes. Experimental outcomes showed that the administration of these inhibitors led to differential reduction in IL-1β, IL-6 (pro-inflammatory), and IL-10 (anti-inflammatory) cytokine production. These findings suggested that inhibition of EP2 and EP4 receptors can modulate cytokine secretion in *S. aureus*-induced dairy cow neutrophils.

Additionally, we investigated the way in which EP2 or EP4 receptor suppression affected initiation of intracellular signaling cascades after neutrophil stimulation by *S. aureus*. The results indicated that both inhibitors decreased the phosphorylation intensities of ERK, P38, and P65 kinases. Cumulatively, the evidence supports that PGE_2_ facilitated NF-κB and MAPK pathway stimulation through EP2/EP4 receptor mechanisms, consequently governing the dual regulation of inflammatory and anti-inflammatory cytokine dynamics. This conclusion is consistent with our previous findings relating to mouse peritoneal macrophages [44,45,46].

Finally, to investigate whether the PGE_2_-EP2/EP4 receptor pathway influenced the phagocytic function of bovine neutrophils induced by *S. aureus*, bovine neutrophils were pretreated with COX-2 and mPGES-1 inhibitors as well as EP2 and EP4 receptor antagonists. The phagocytic capacity of these cells against *S. aureus* was assessed. The results indicated that the PGE_2_-EP2/EP4 receptor pathway did not significantly affect the phagocytic function of bovine neutrophils induced by *S. aureus*. Wu et al. (2020) similarly reported that inhibition of COX-2 and mPGES-1 in mouse peritoneal macrophages resulted in negligible changes in their phagocytic functionality [39]. Experiments conducted on human neutrophils have demonstrated that exogenously administered PGE_2_ significantly inhibits neutrophil phagocytosis of *E. coli* via the EP2R-cAMP-PTEN signaling pathway [9]. These studies indicate that the exogenous addition of PGE_2_ inhibits neutrophil phagocytosis, whereas a reduction in endogenous PGE_2_ secretion does not influence the cells’ phagocytic capacity against *S. aureus.* It should be noted, however, that neutrophils exhibit variable responses to bacteria of different species, and even among various strains of the same species [42,43]. Therefore, further research is warranted to investigate the effects of COX-2 and mPGES-1 inhibitors, as well as EP2 and EP4 receptor antagonists, on neutrophil phagocytic function in response to different bacterial species. Such studies would provide valuable experimental evidence for expanding their potential clinical applications. Meanwhile, the activation state of neutrophils prior to the experiment, as well as other potential confounding variables, may influence the experimental outcomes. To address this, we minimized such variability in our study by increasing the number of donor dairy cows, strictly monitoring the health status of the donor dairy cows, assessing cell viability, and repeating the experiments under consistent conditions.

In conclusion, the endogenous PGE_2_-EP2/4 receptor pathway exerts a certain regulatory influence on the inflammatory response of dairy cow neutrophils induced by *S. aureus*, without affecting the pathogen clearance capacity of these cells. These findings identify mPGES-1 and the EP2 and EP4 receptors as potential therapeutic targets for anti-inflammatory interventions. The anti-inflammatory efficacy of inhibitors targeting these molecules appears comparable to that of traditional non-steroidal anti-inflammatory drugs, such as COX-2 inhibitors, while potentially circumventing the adverse effects associated with conventional therapies. Furthermore, this study demonstrates that the mPGES-1 inhibitor MF63 has potential as a candidate drug for the treatment of diseases in dairy cows. Nevertheless, since this study is confined to in vitro experiments, future in vivo investigations are required to assess the actual efficacy and safety of these inhibitors in dairy cows, thereby laying a foundation for their potential clinical application.

## 5. Conclusions

When *S. aureus* triggers a response in dairy cow neutrophils, intracellular PGE_2_ activates NF-κB and MAPK signaling cascades through EP2/EP4 receptor engagement, modulating the production and release of inflammatory and regulatory cytokines. This dual mechanism orchestrated a balanced immune response through dynamic regulation of cytokine networks. The signaling crosstalk between these pathways demonstrated the complex regulation of inflammatory mediators mediated by prostaglandin receptor interactions.

## Data Availability

The original contributions presented in this study are included in the article/Supplementary Materials. Further inquiries can be directed to the corresponding author.

## Supplementary Materials

The following supporting information can be downloaded at: https://www.mdpi.com/article/10.3390/biom15081062/s1, Table S1 The Physical Health Status of Donor Cows.

## Abbreviations

The following abbreviations are used in this manuscript:

AA	Arachidonic acid
AH23848	EP4 receptor blocker
ANOVA	Analysis of variance
CAY10404	Cyclooxygenase-2 suppressor
COX-2	Cyclooxygenase-2
ELISA	Enzyme-linked immunosorbent assay
ERK	Extracellular signal-regulated kinase
mPGES-1	Microsomal prostaglandin E synthase-1
MF63	Microsomal prostaglandin E synthase-1 inhibitor
NSAIDs	Non-steroidal anti-inflammatory medications
PGE_2_	Prostaglandin E_2_
