# Supplementary material for: Regulatory Effect of PGE2-EP2/EP4 Receptor Pathway on Staphylococcus aureus-Induced Inflammatory Factors in Dairy Cow Neutrophils
Source: Biomolecules. 2025 Jul 22;15(8):1062. doi: 10.3390/biom15081062 (PMC12383739; doi:10.3390/biom15081062)
Supplement: Supplementary file 1 [file biomolecules-15-01062-s001.zip › biomolecules-3709263-original-images.pdf]

Fig 2 +cay10404

ERK

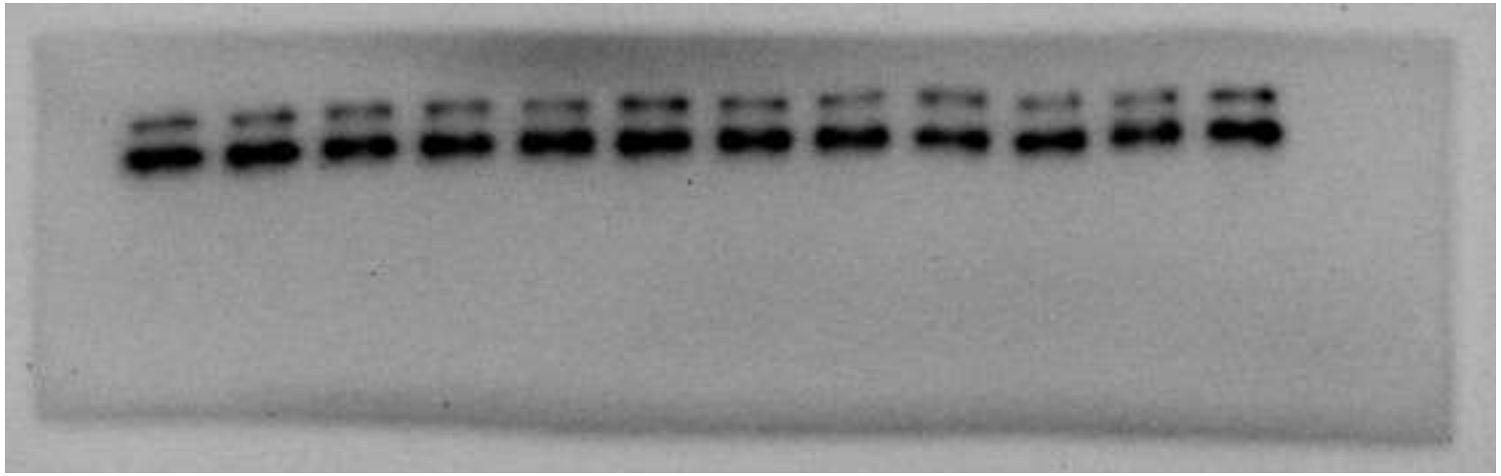

42,44 kDa

P-ERK

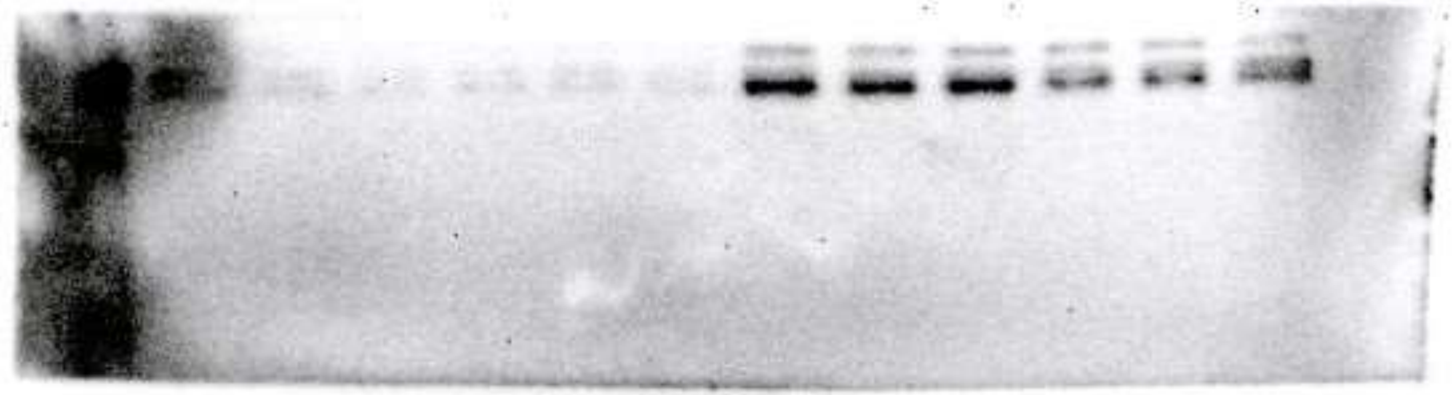

42,44 kDa

Fig 2 +cay10404

P38

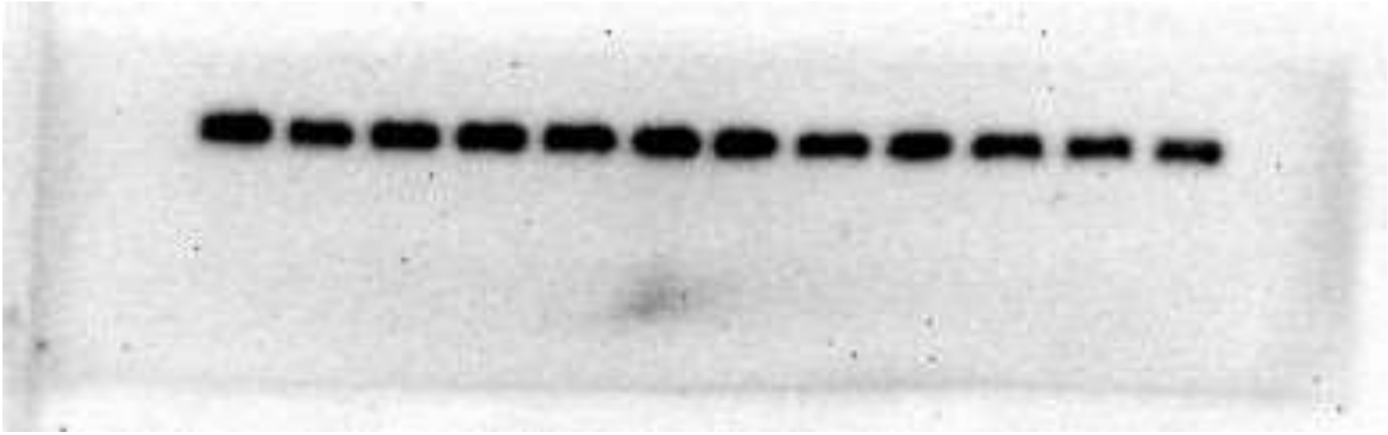

38 kDa

P-P38

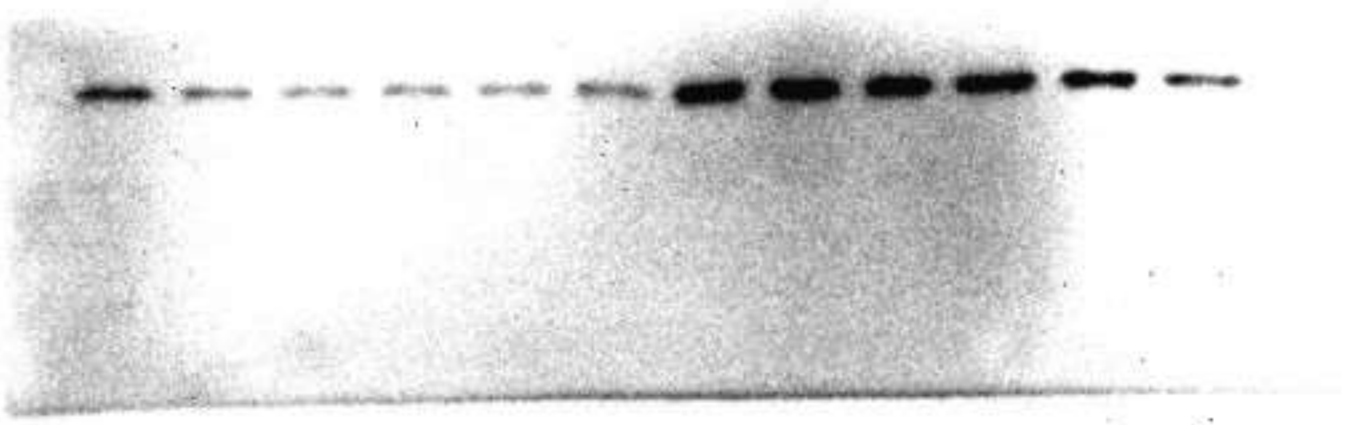

38 kDa

Fig 2 +cay10404

P65

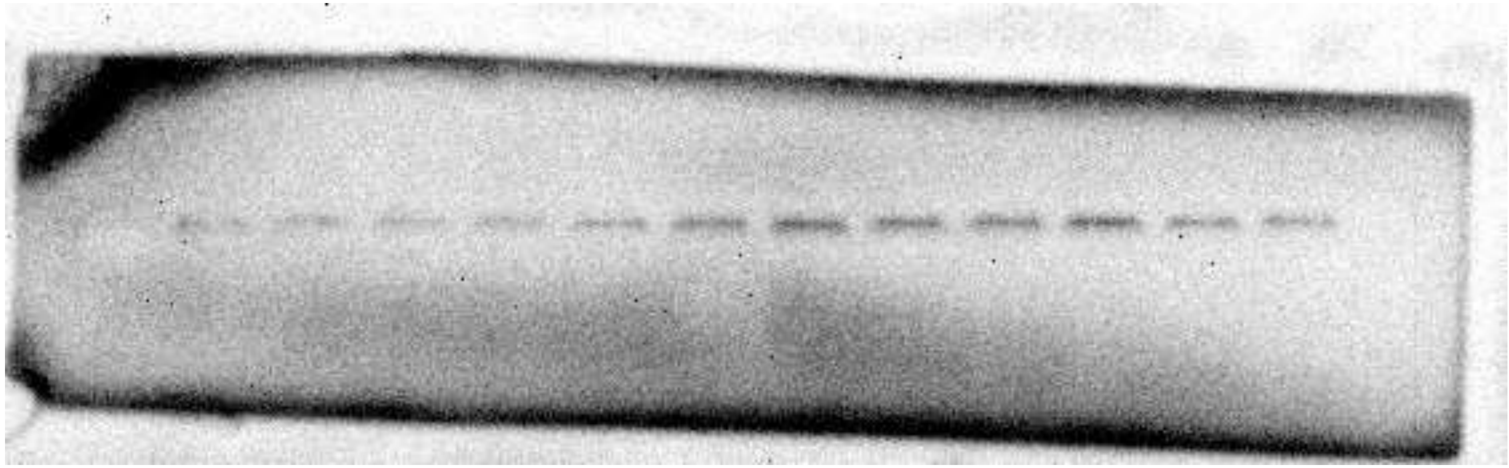

65 kDa

P-P65

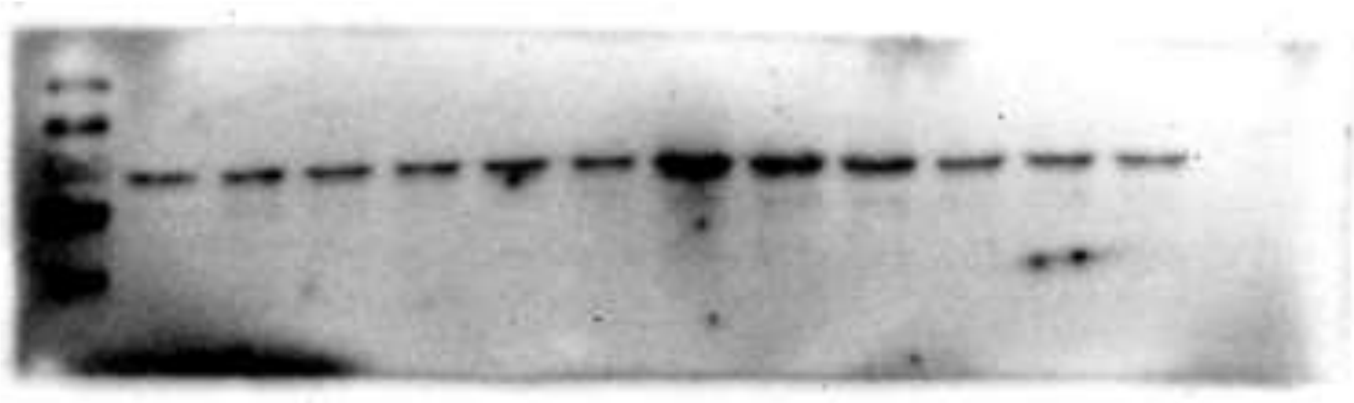

65 kDa

Fig 2 +cay10404

GAPDH

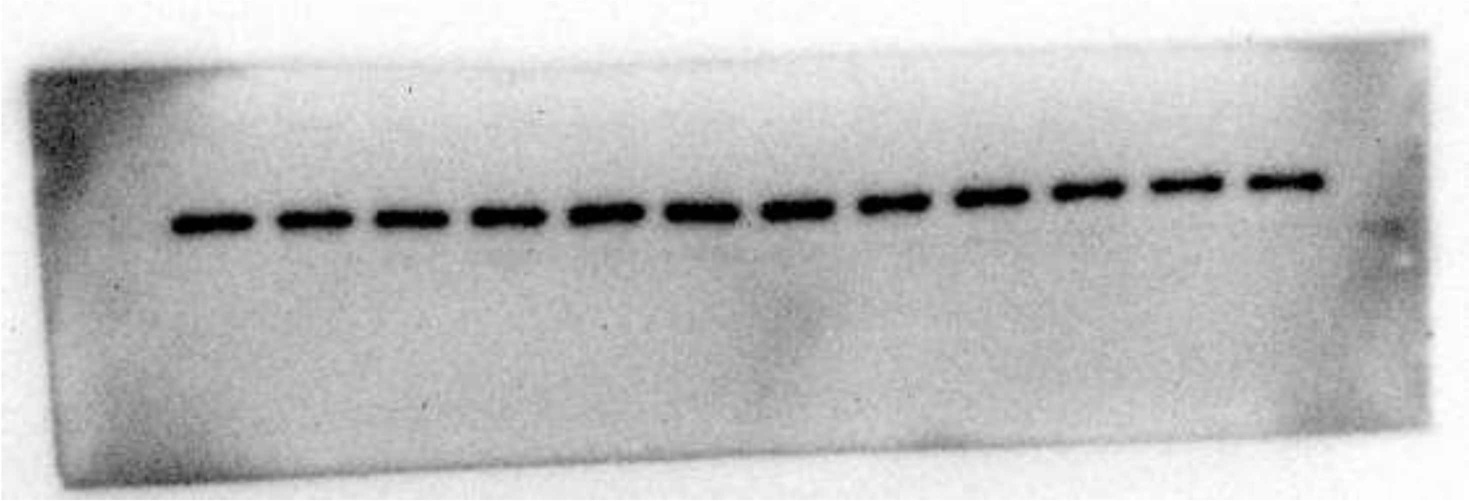

37 kDa

Fig 2 +mf63

ERK

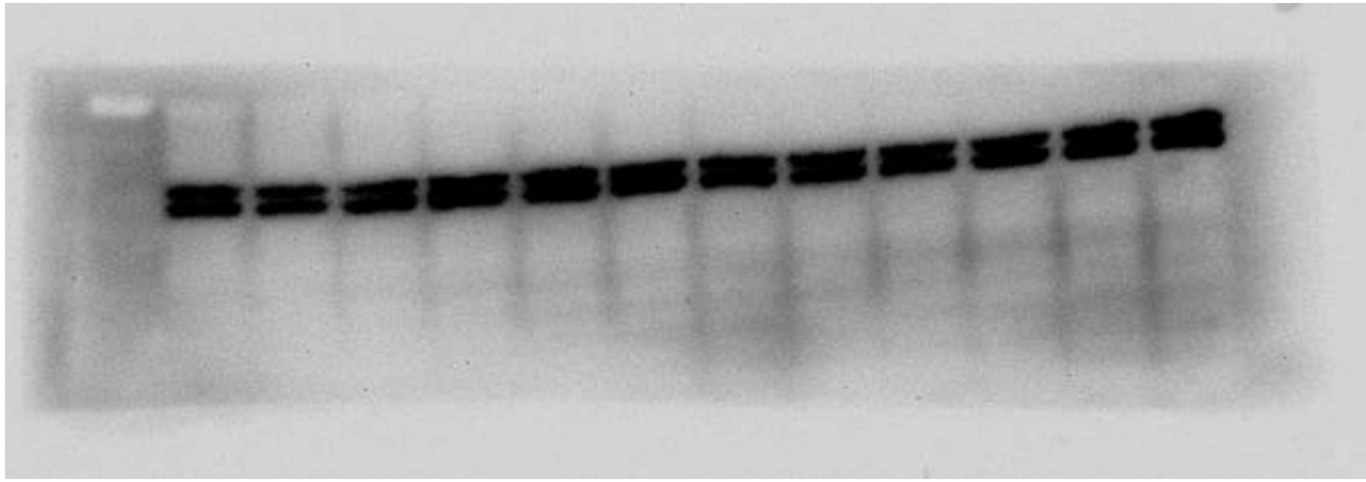

42,44 kDa

P-ERK

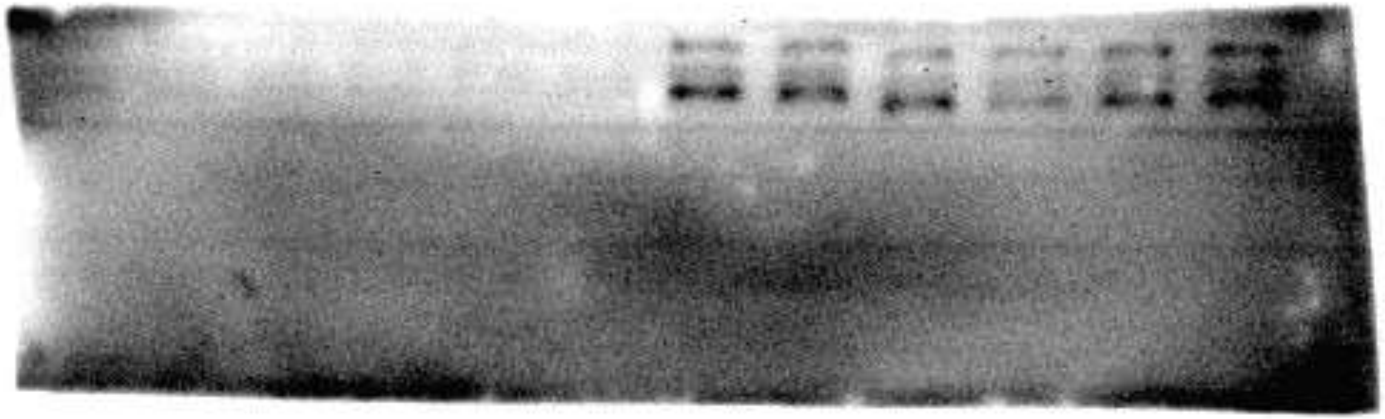

42,44 kDa

Fig 2 +mf63

P38

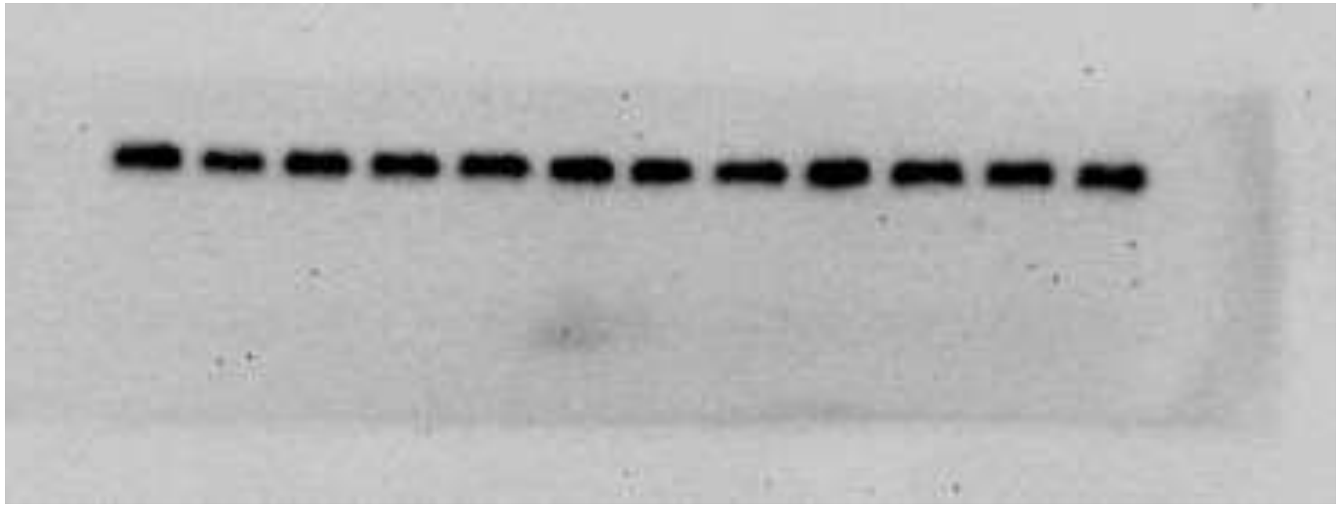

38 kDa

P-P38

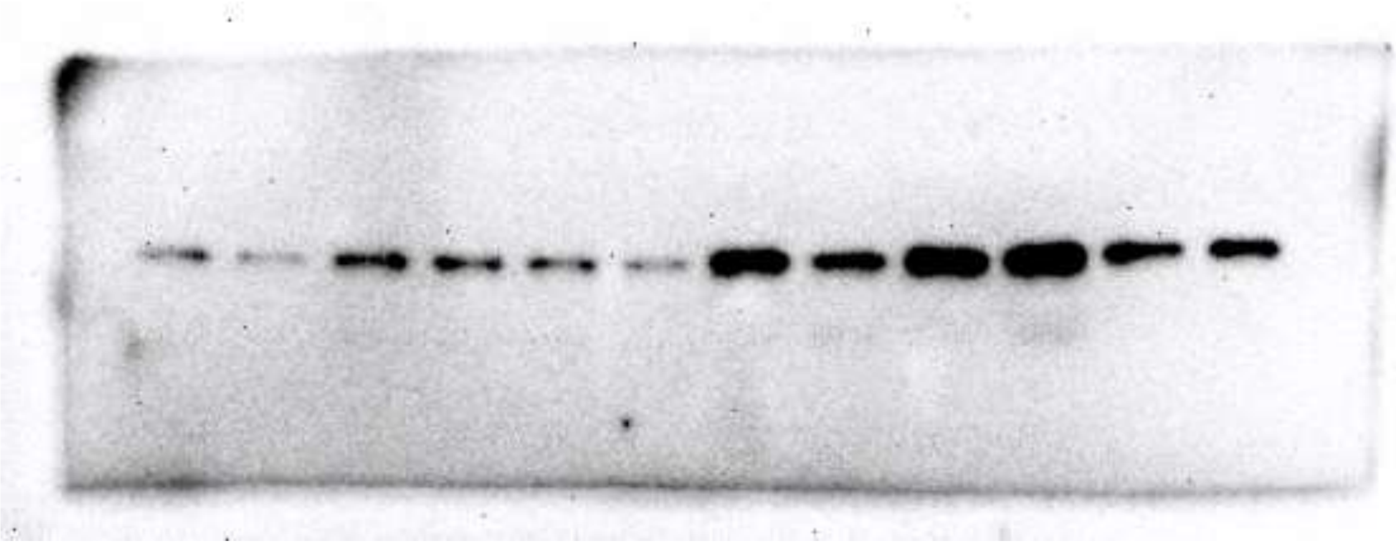

38 kDa

Fig 2 +mf63

P65

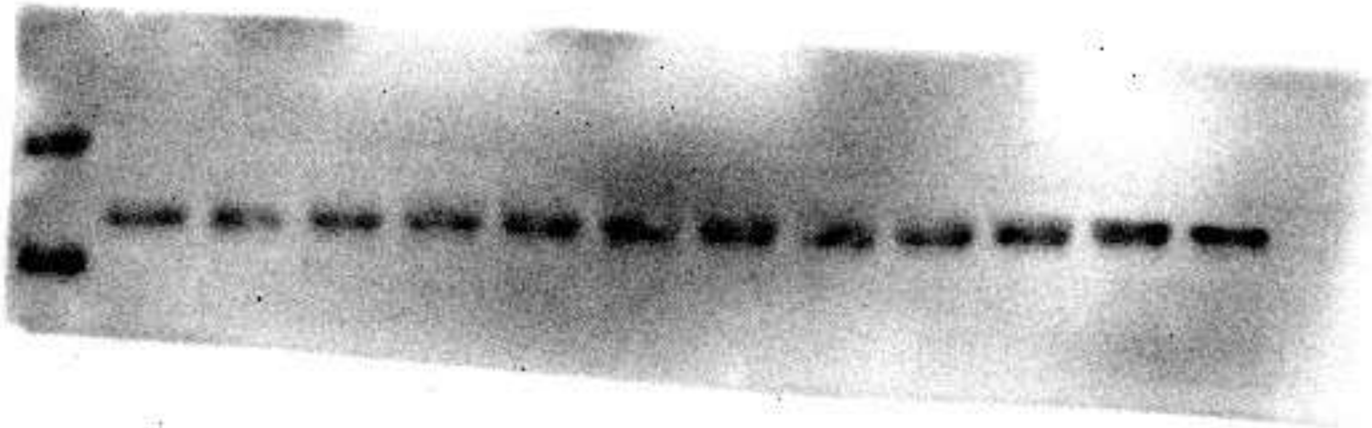

65 kDa

P-P65

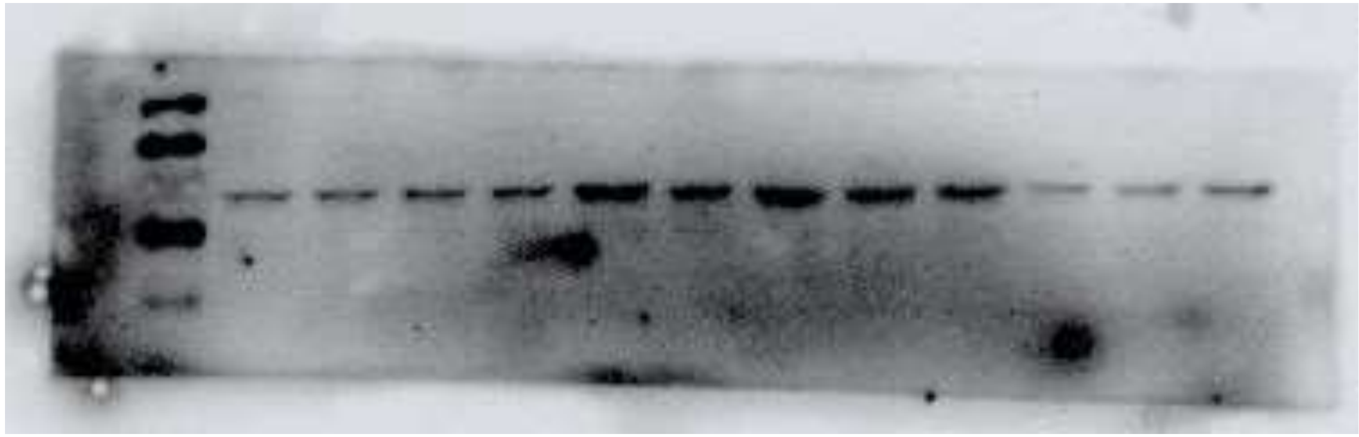

65 kDa

Fig 2 +mf63

GAPDH

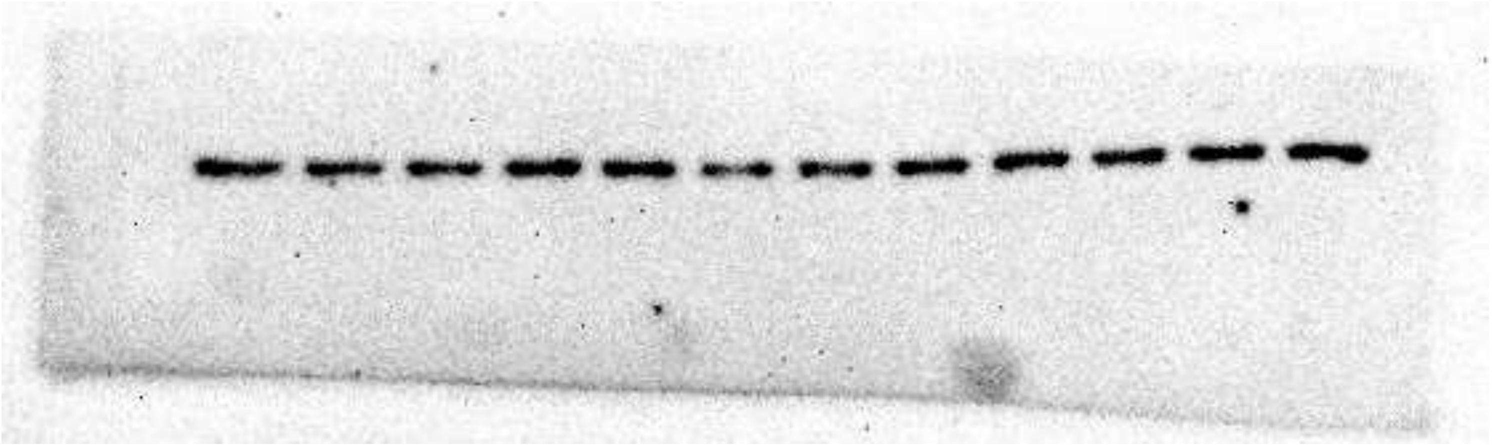

37 kDa

Fig 4 +AH6809

ERK

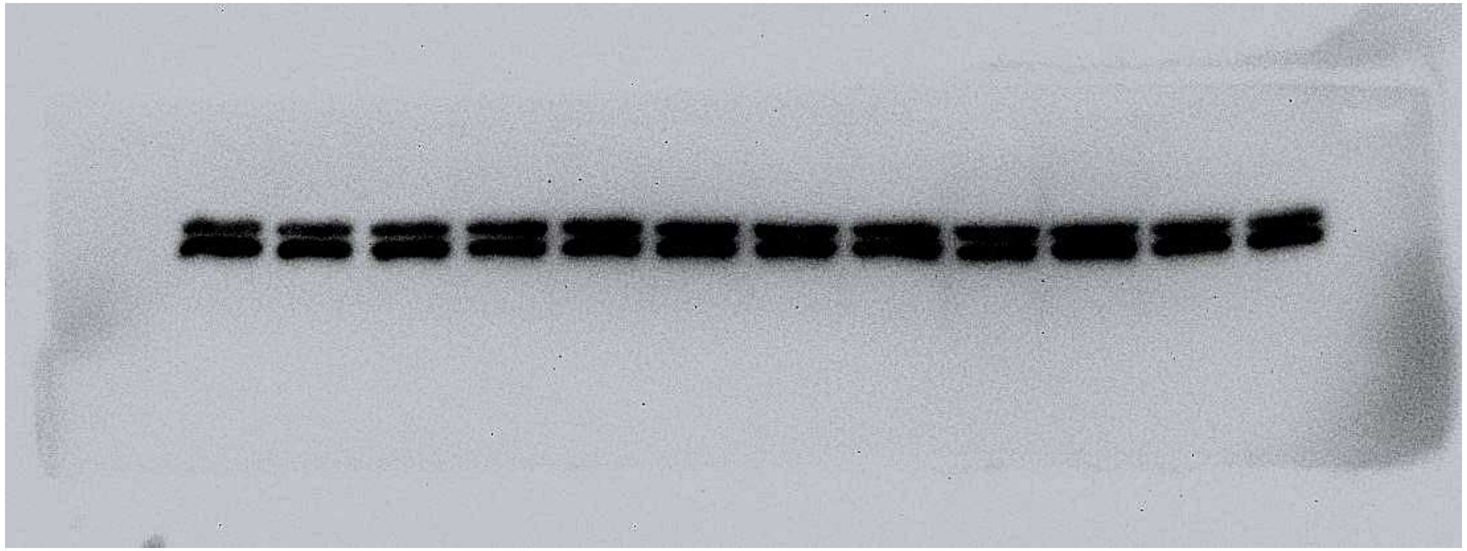

42,44 kDa

P-ERK

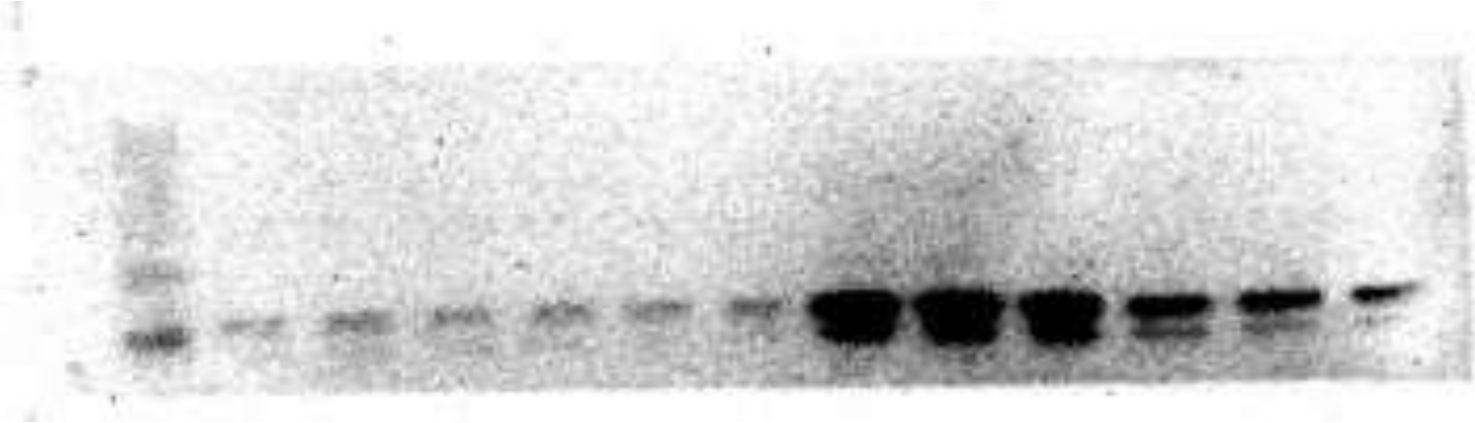

42,44 kDa

Fig 4 +AH6809

P38

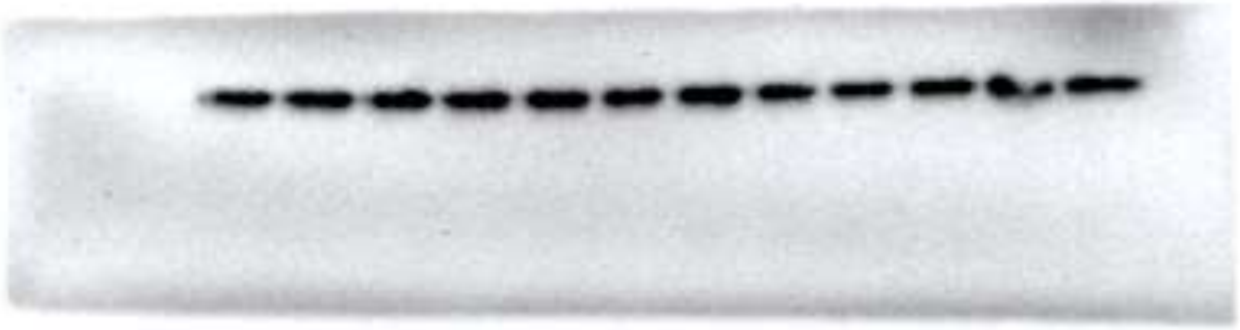

38 kDa

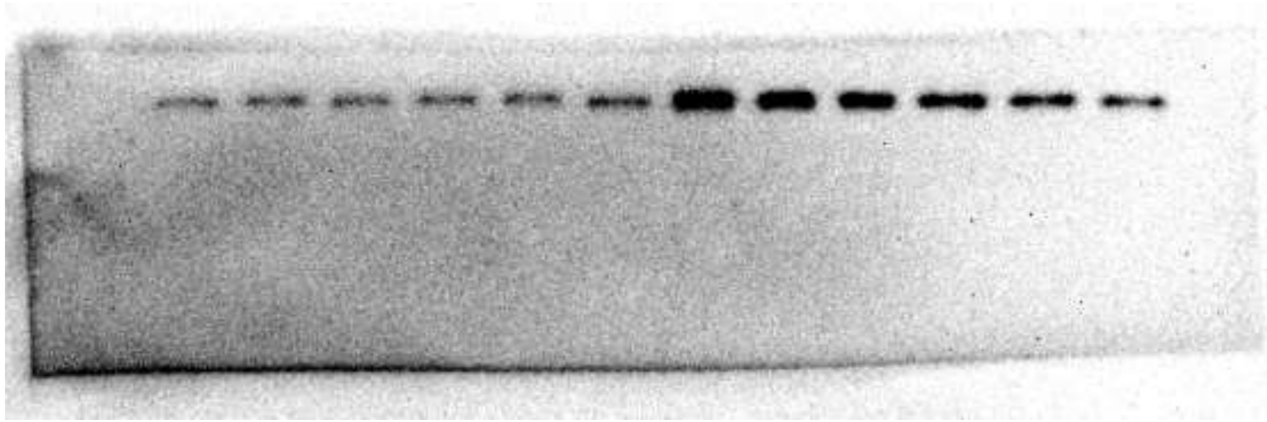

P-P38

38 kDa

Fig 4 +AH6809

P65

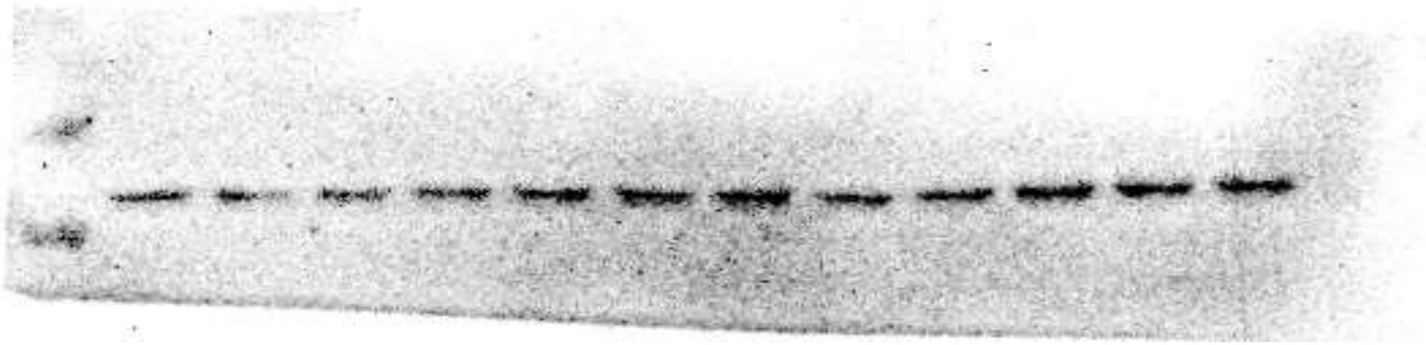

65 kDa

P-P65

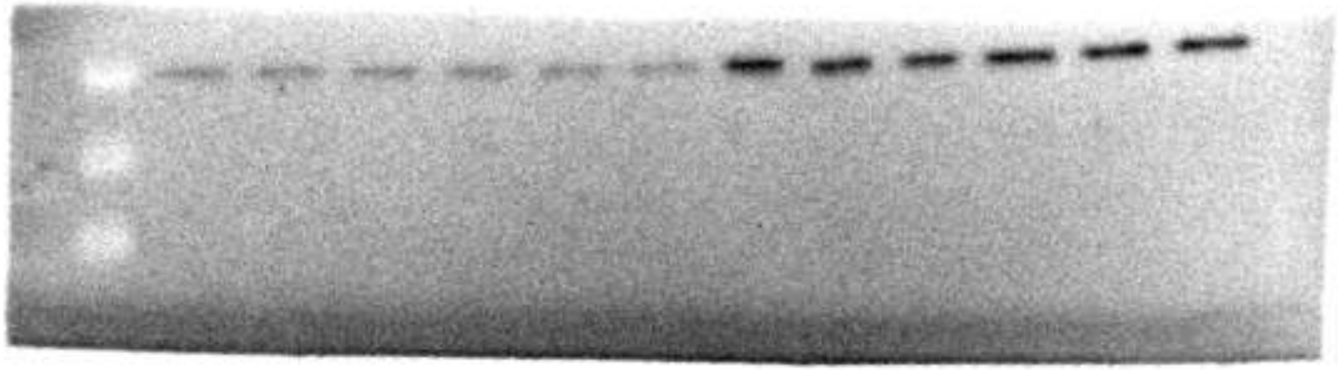

65 kDa

Fig 4 +AH6809

GAPDH

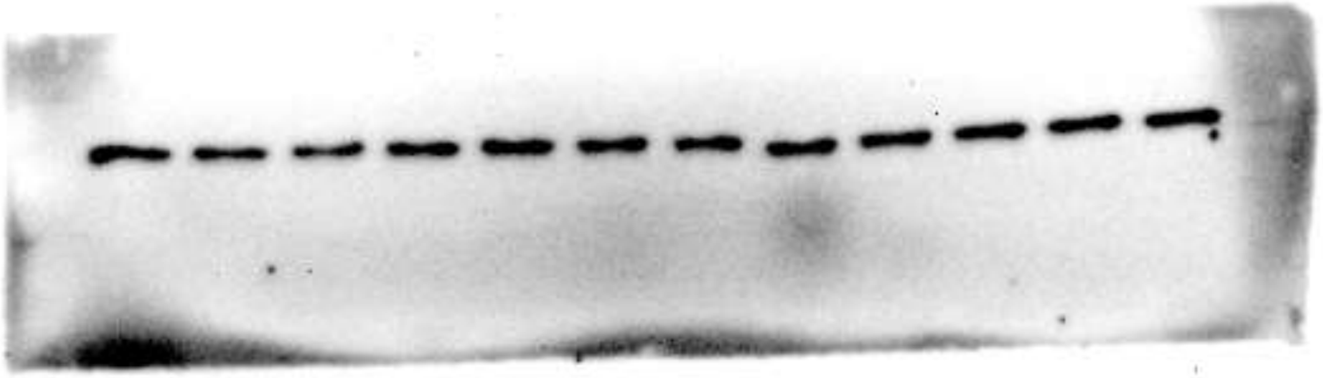

37 kDa

Fig 4 +AH23848

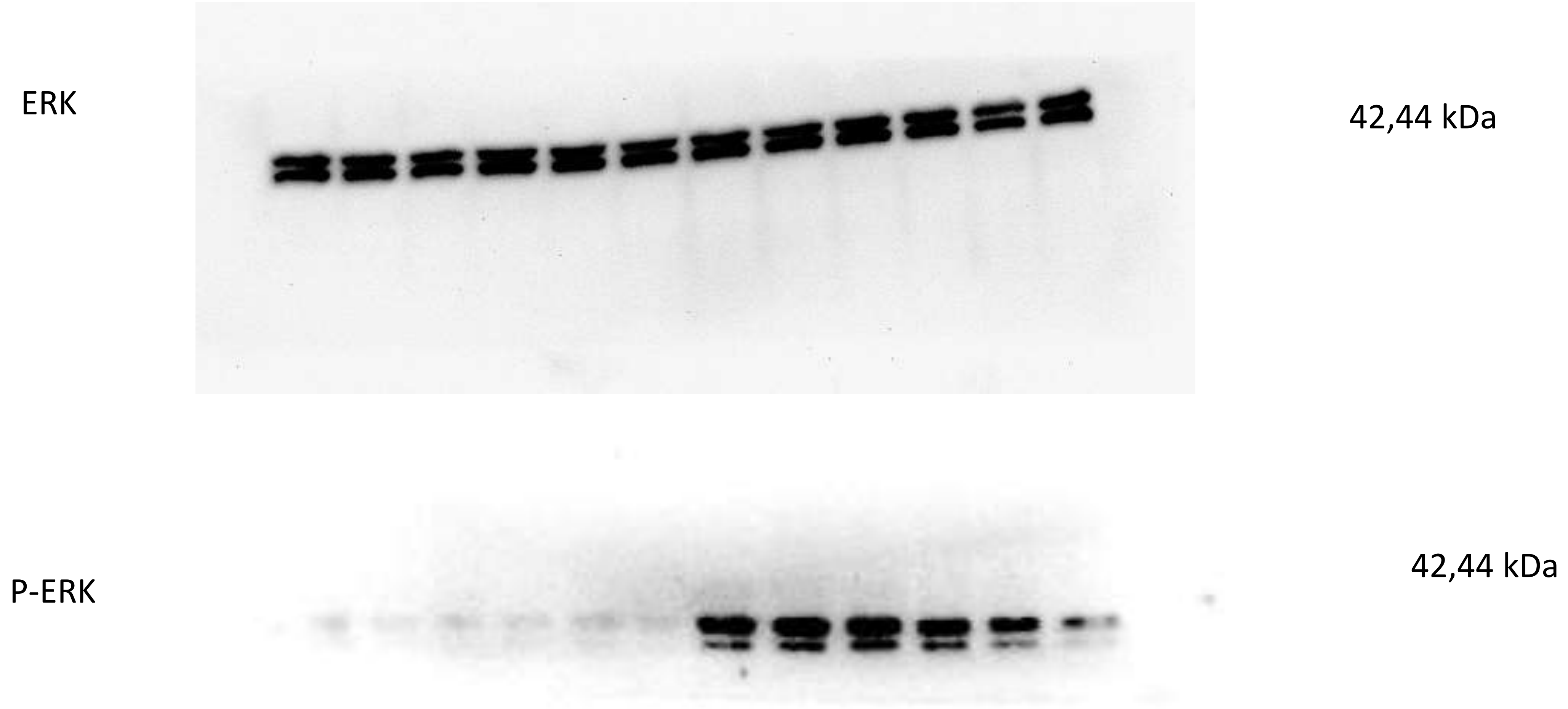

Fig 4 +AH23848

P38

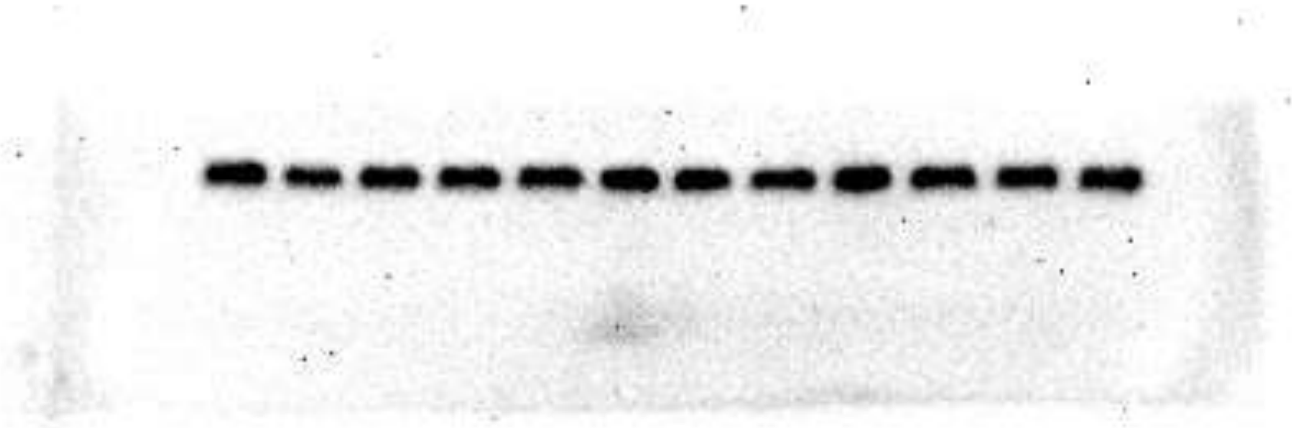

38 kDa

P-P38

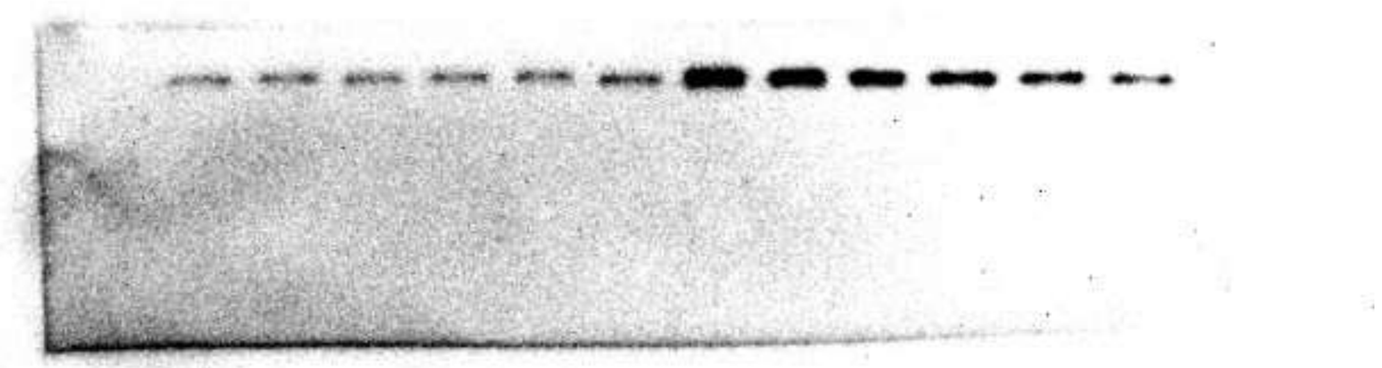

38 kDa

Fig 4 +AH23848

P65

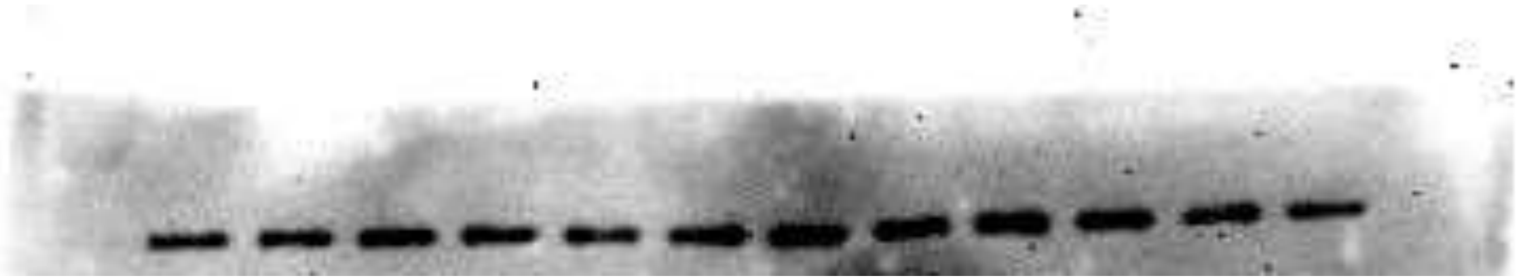

65 kDa

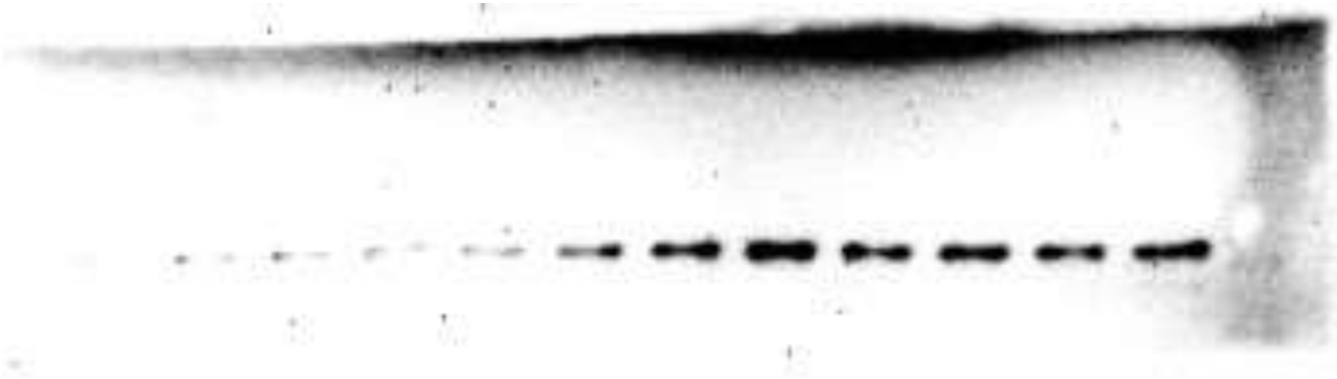

P-P65

65 kDa

Fig 4 +AH23848

GAPDH

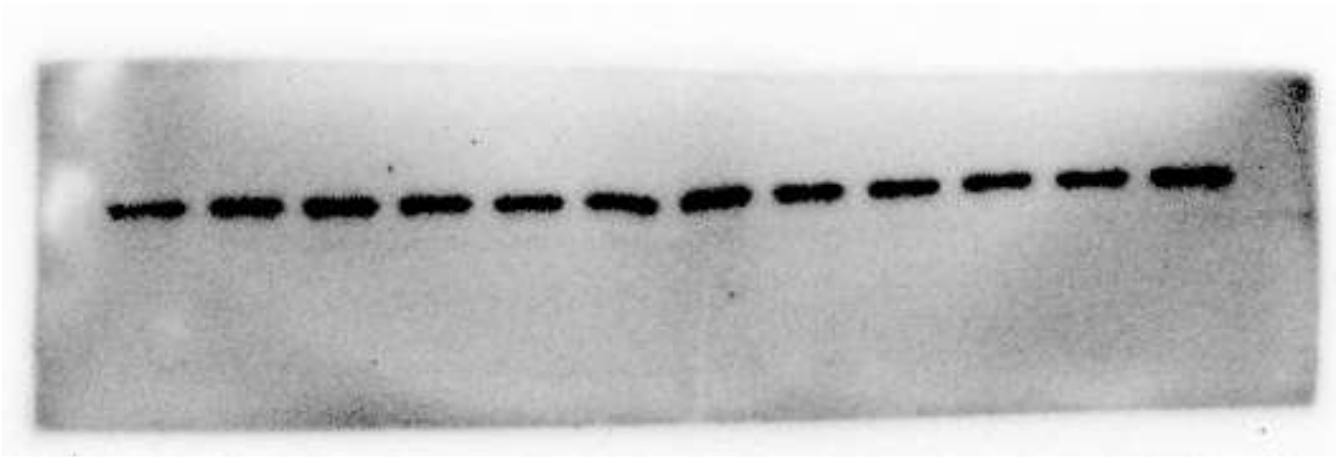

37 kDa
