# Supplementary material for: Regulatory Effect of PGE2-EP2/EP4 Receptor Pathway on Staphylococcus aureus-Induced Inflammatory Factors in Dairy Cow Neutrophils
Source: Biomolecules. 2025 Jul 22;15(8):1062. doi: 10.3390/biom15081062 (PMC12383739; doi:10.3390/biom15081062)
Supplement: Supplementary file 1 [file biomolecules-15-01062-s001.zip › biomolecules-3709263-supplementary.pdf]

Supplementary material:
Table S1 The Physical Health Status of Donor Cows

**Experimental animal numbering**	**1**	**2**	**3**
**Breed**	Holstein	Holstein	Holstein
**Age (months)**	32	35	34
**Parity**	1	1	1
**Body weight (kg)**	675.33±2.52	691.67±1.528	683.33±1.53
**Day in Milk (d)**	140	135	142
**Dry matter intake（kg/d）**	25.67±4.93	30.00±1	27.67±1.16
**Daily milk yield（kg/d）**	25.66±1.53	28.33±0.58	26.33±0.58
**Body condition score (Edmonson)**	4.25±0.25	4.25±0.25	4.25±0.25
**Health condition**	Healthy	Healthy	Healthy

Fig 2 +CAY10404

ERK

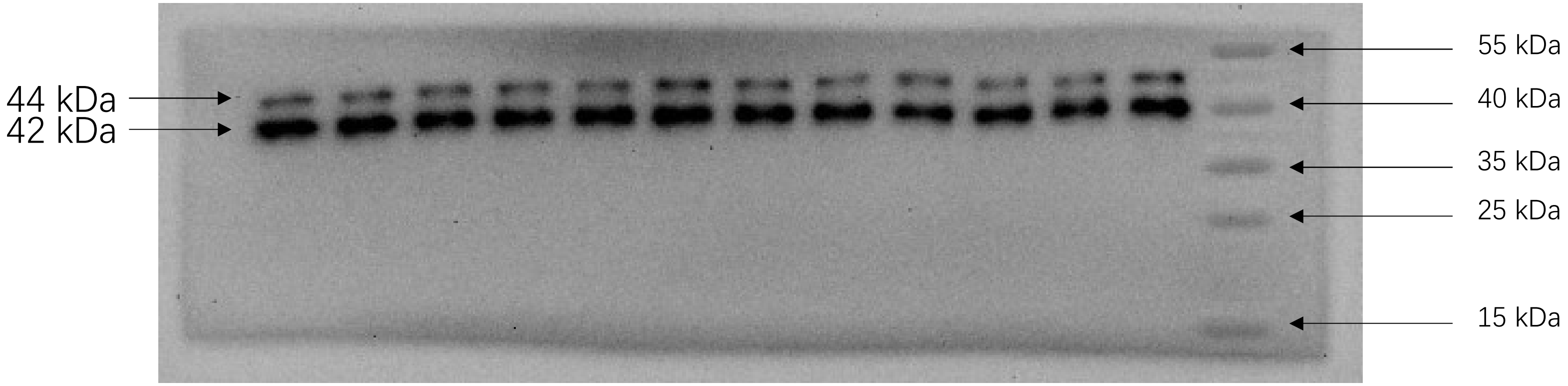

P-ERK

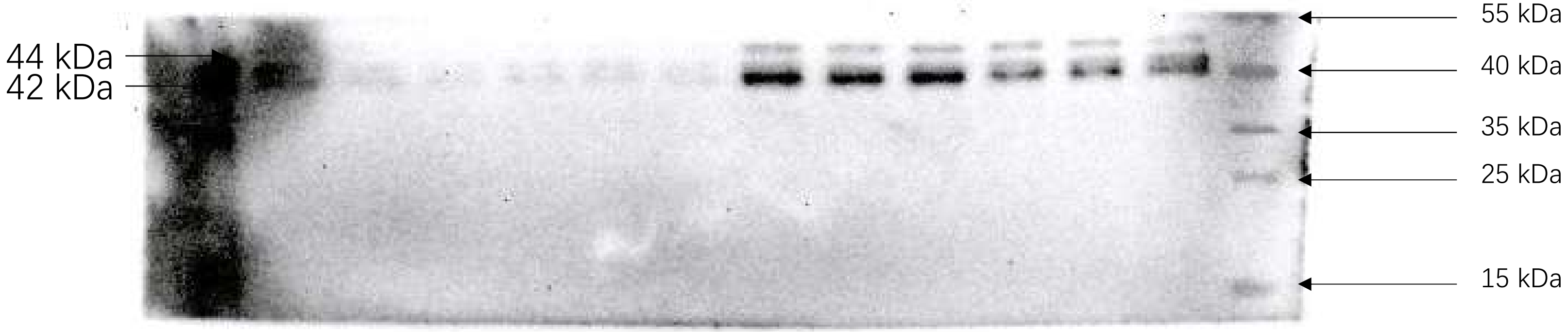

Fig 2 +CAY10404

P38

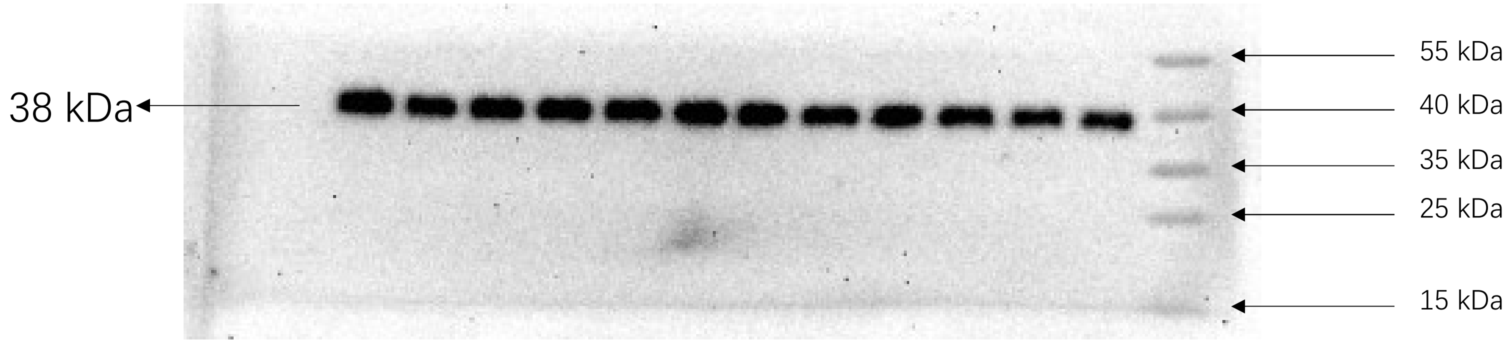

p-P38

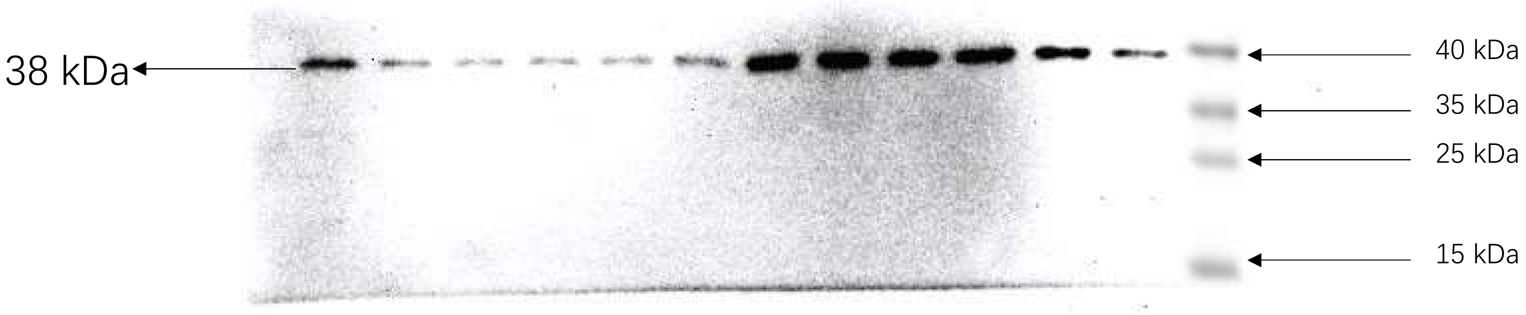

Fig 2 +CAY10404

P65

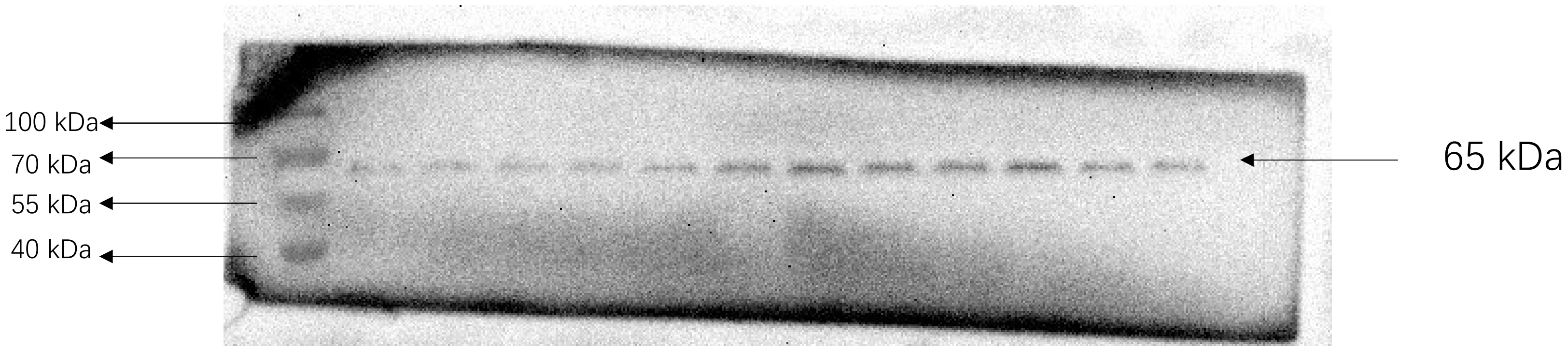

p-P65

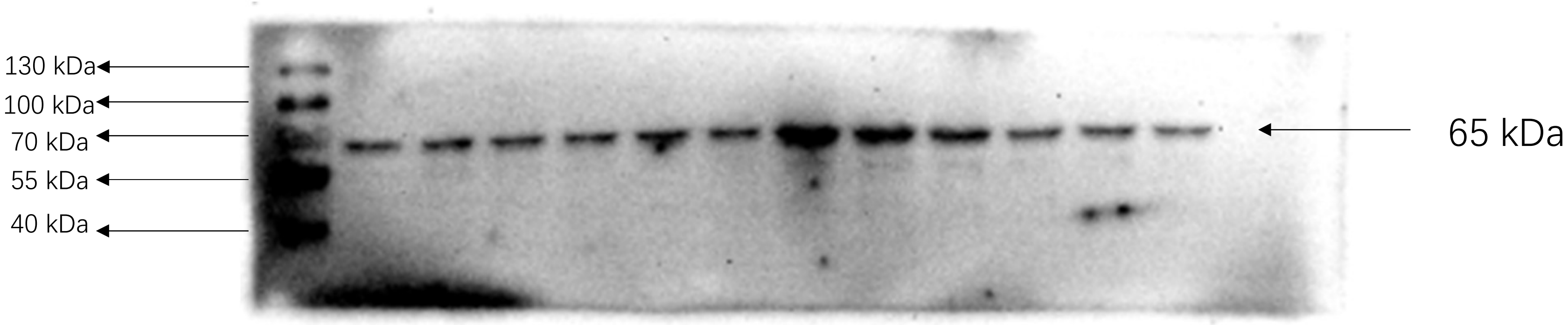

Fig 2 +CAY10404

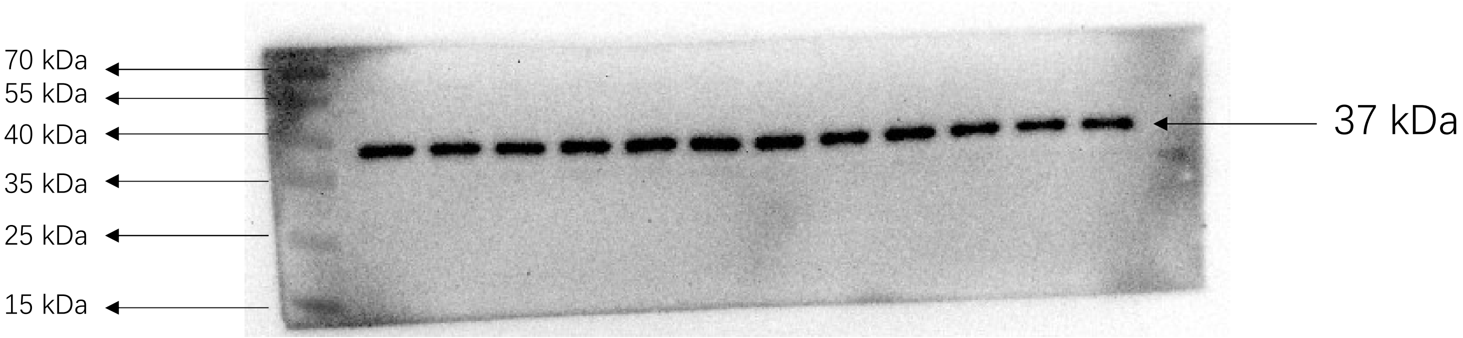

GAPDH

Fig 2 + MF63

ERK

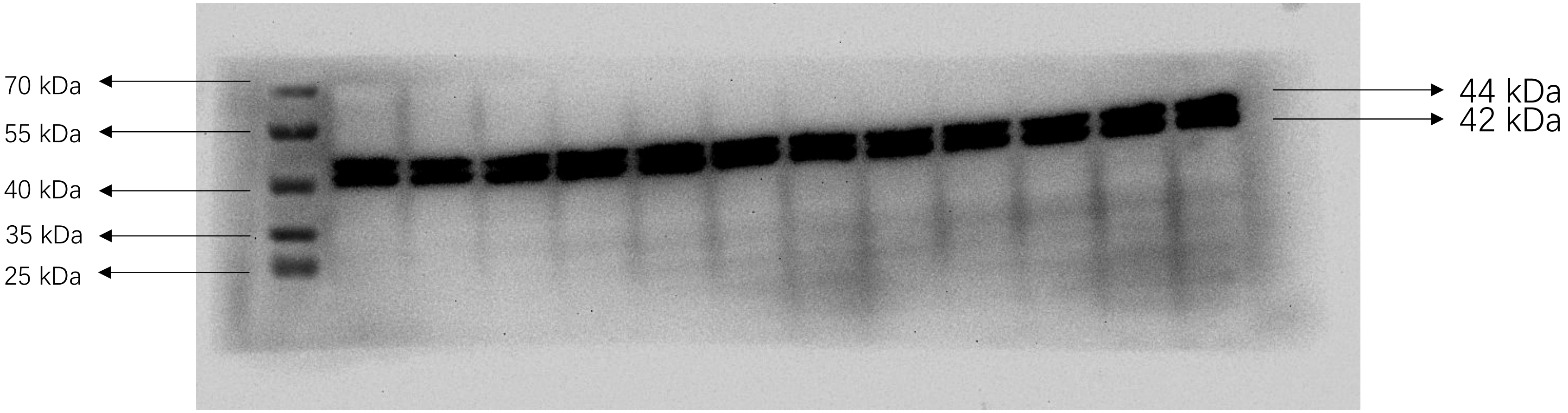

P-ERK

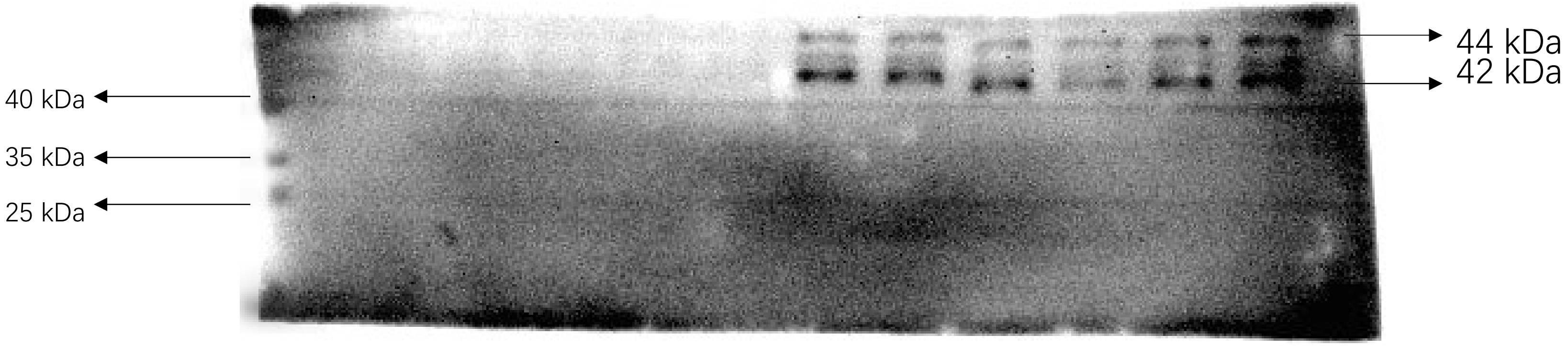

Fig 2 + MF63

P38

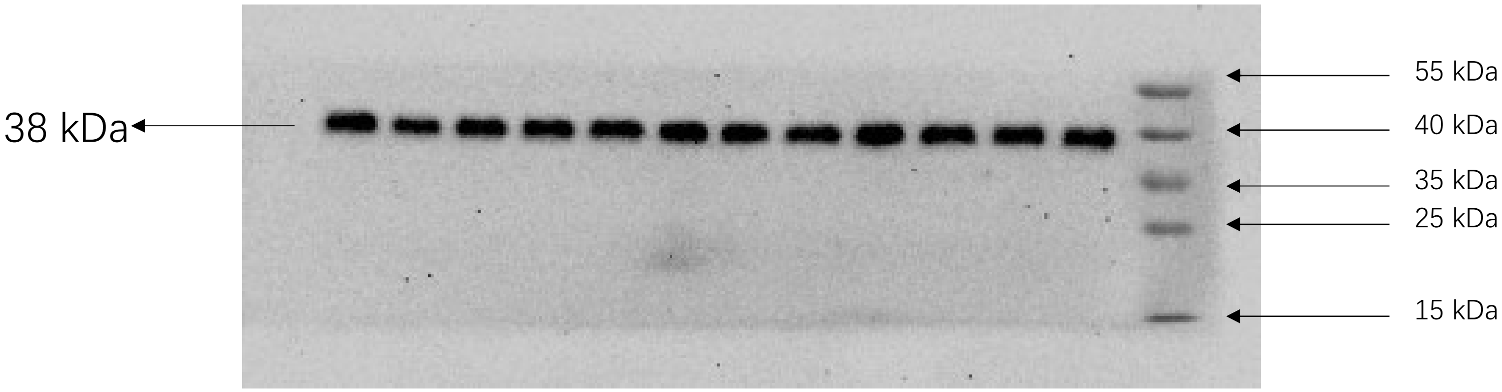

p-P38

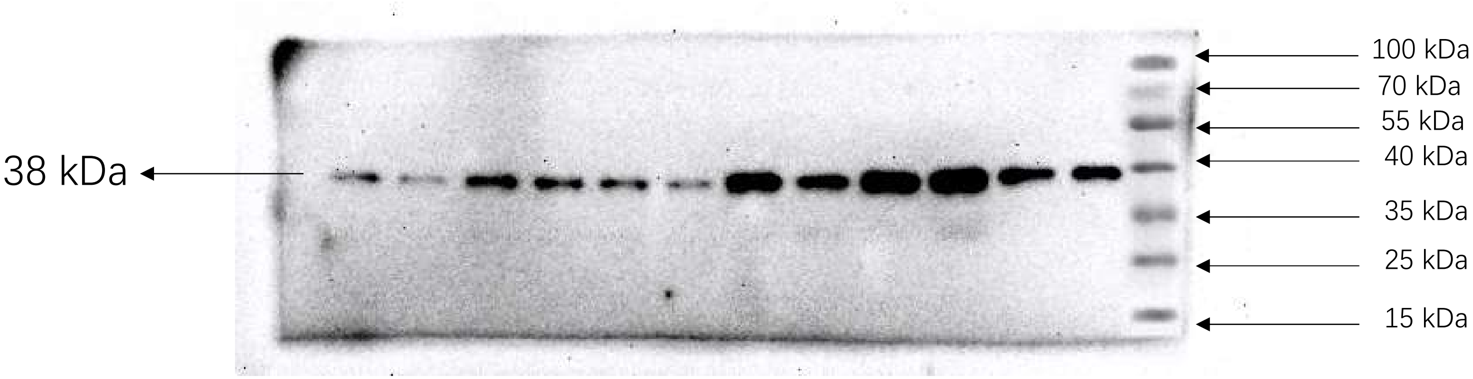

Fig 2 +MF63

P65

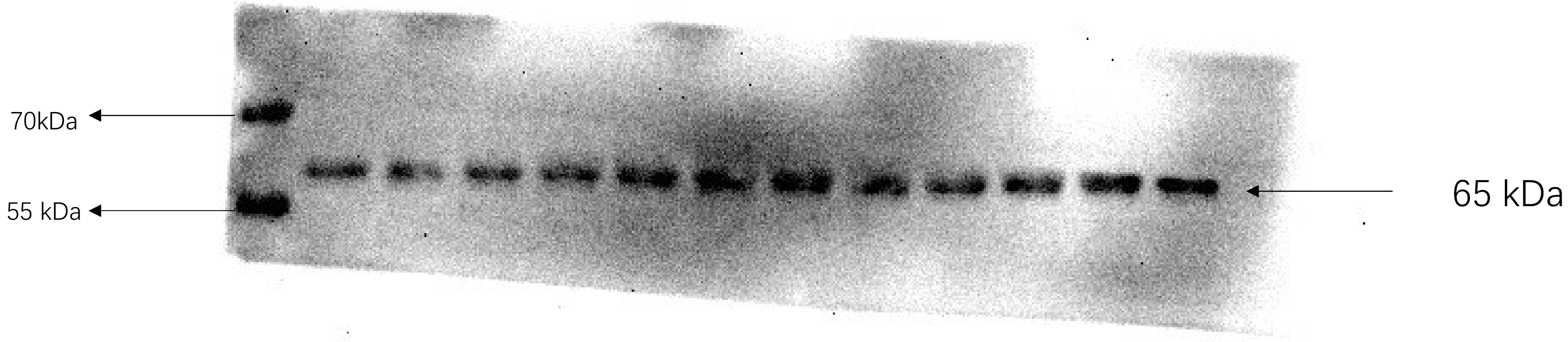

P-P65

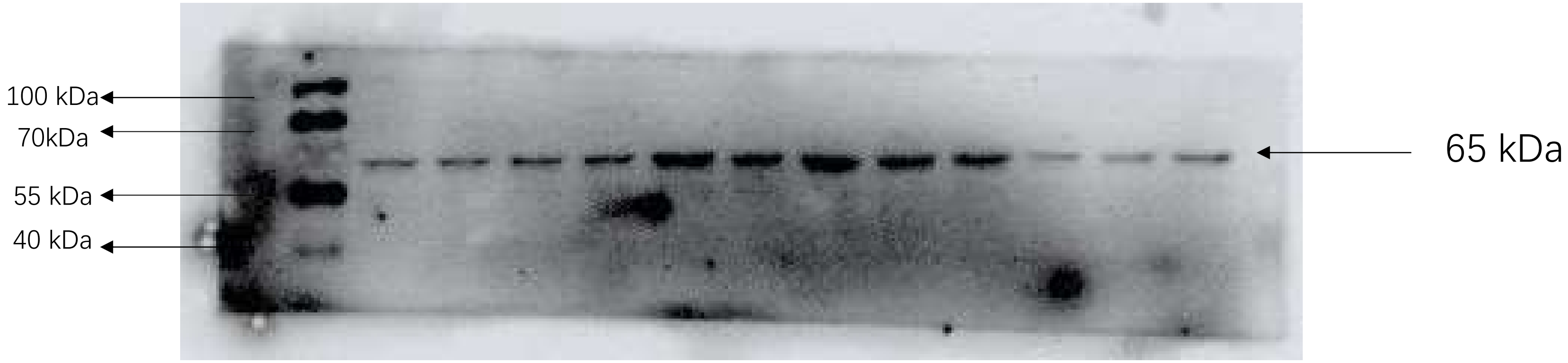

Fig 2 + MF63

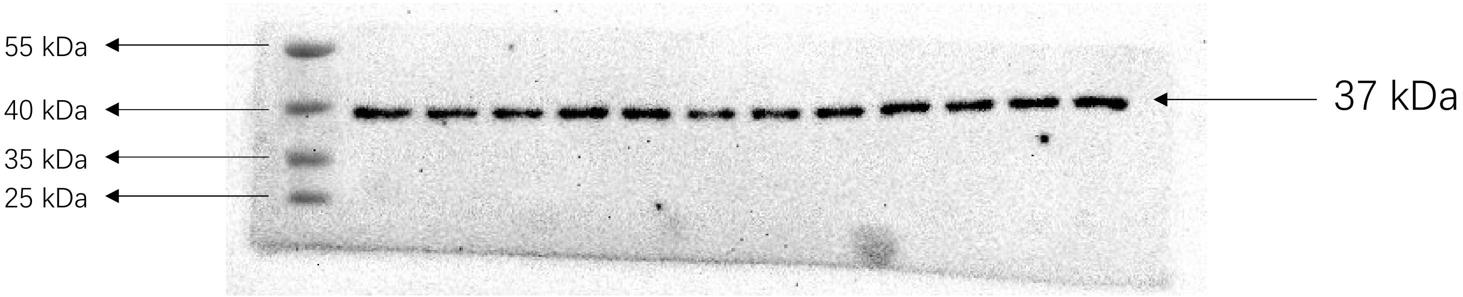

GAPDH

Fig 4 +AH6809

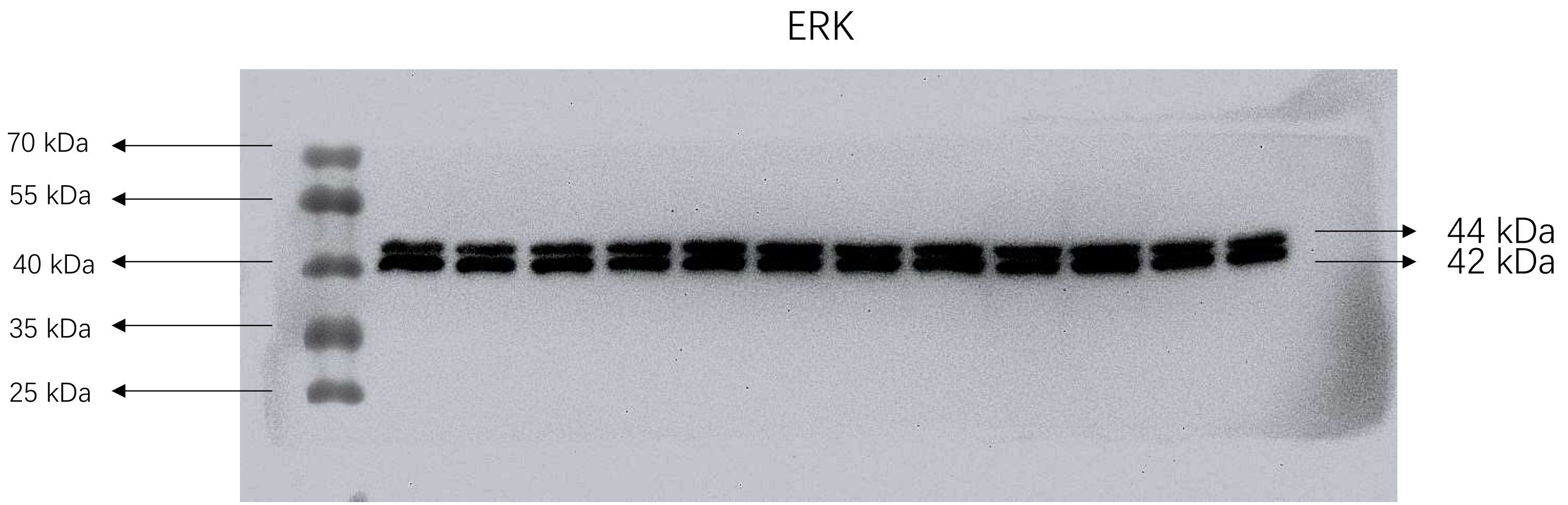
ERK
70 kDa
55 kDa
40 kDa
35 kDa
25 kDa
44 kDa
42 kDa

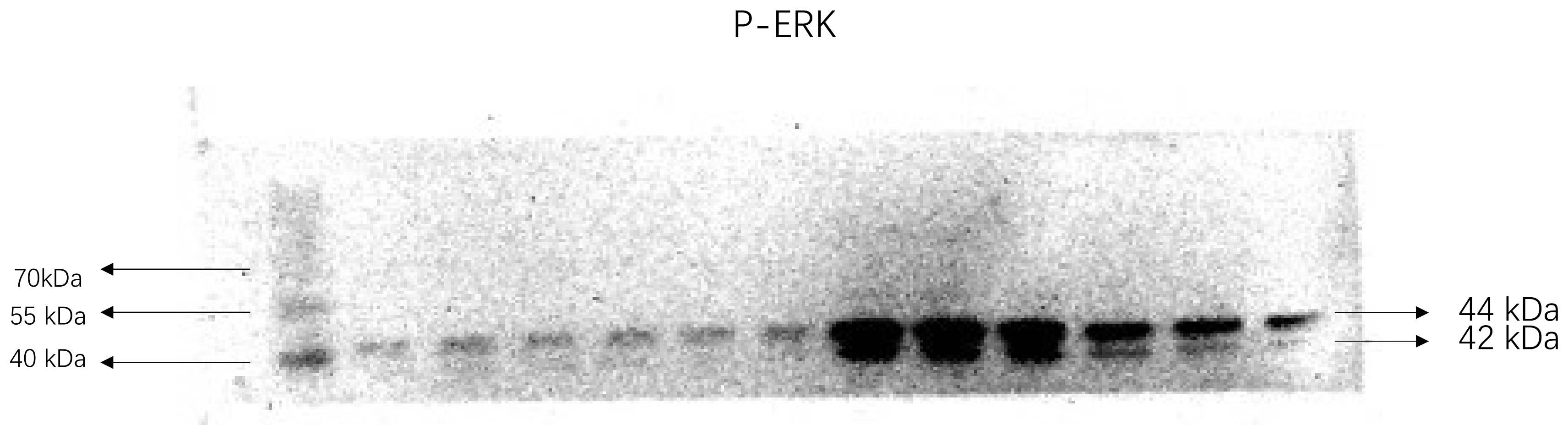
P-ERK
70kDa
55 kDa
40 kDa
44 kDa
42 kDa

Fig 4 +AH6809

P38

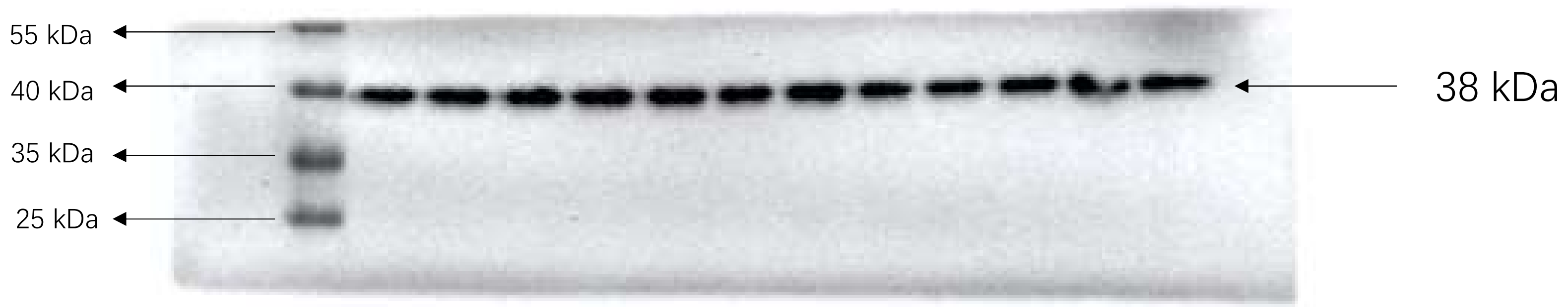

p-P38

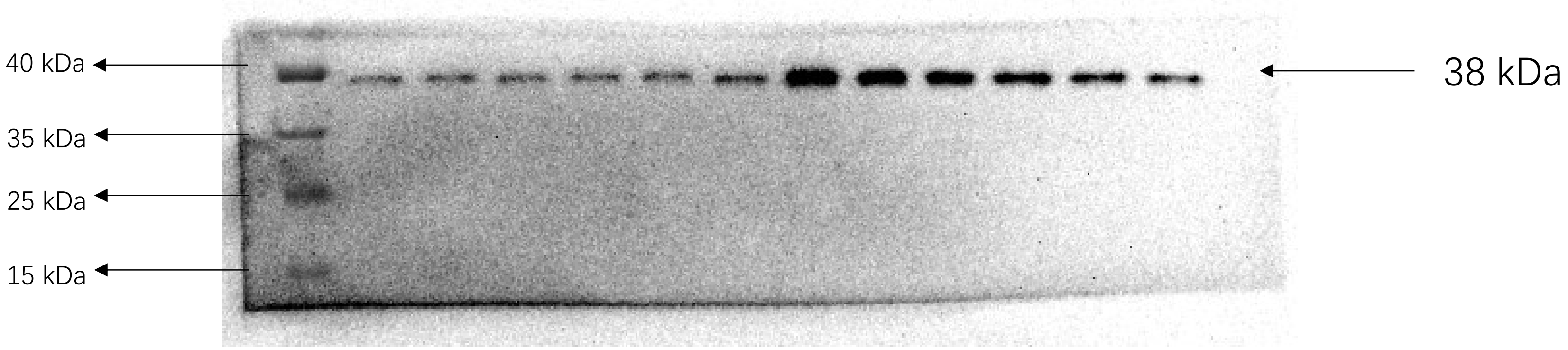

Fig 4 +AH6809

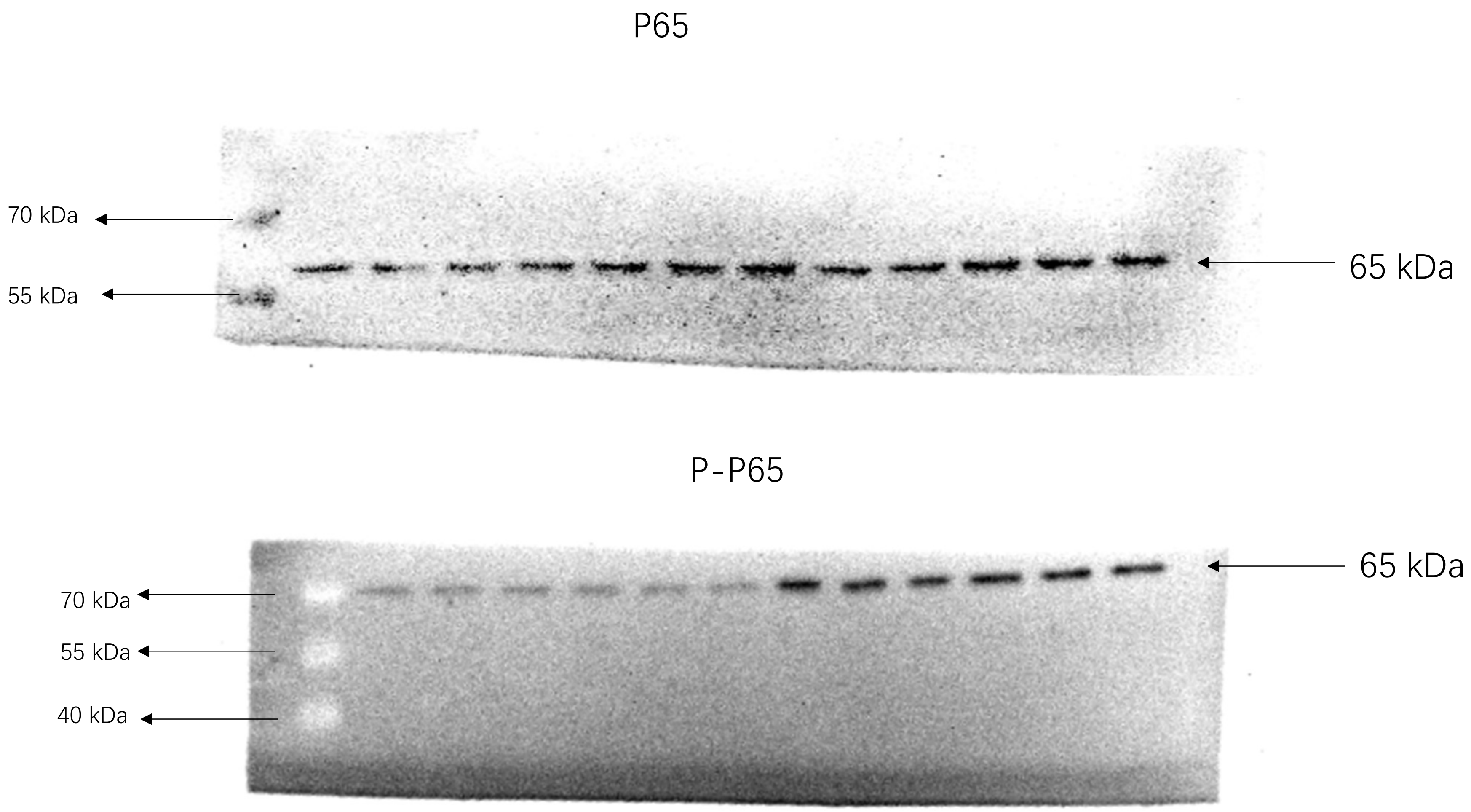

Fig 4 +AH6809

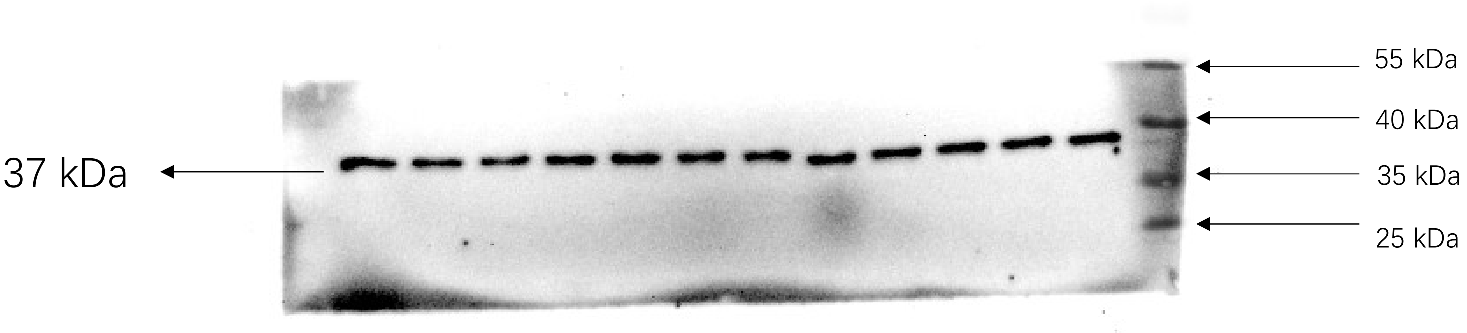

GAPDH

Fig 4 +AH23848

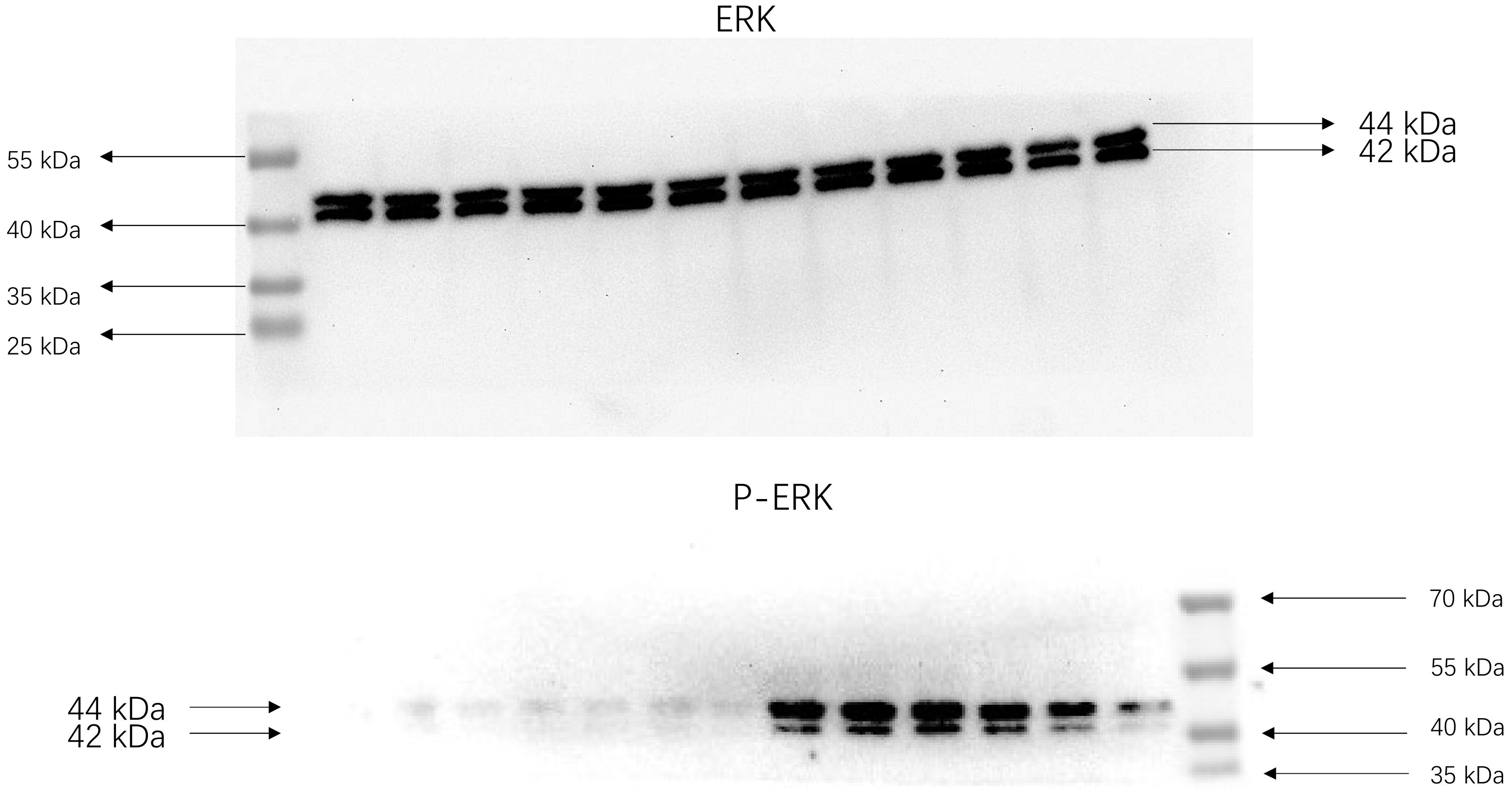

Fig 4 +AH23848

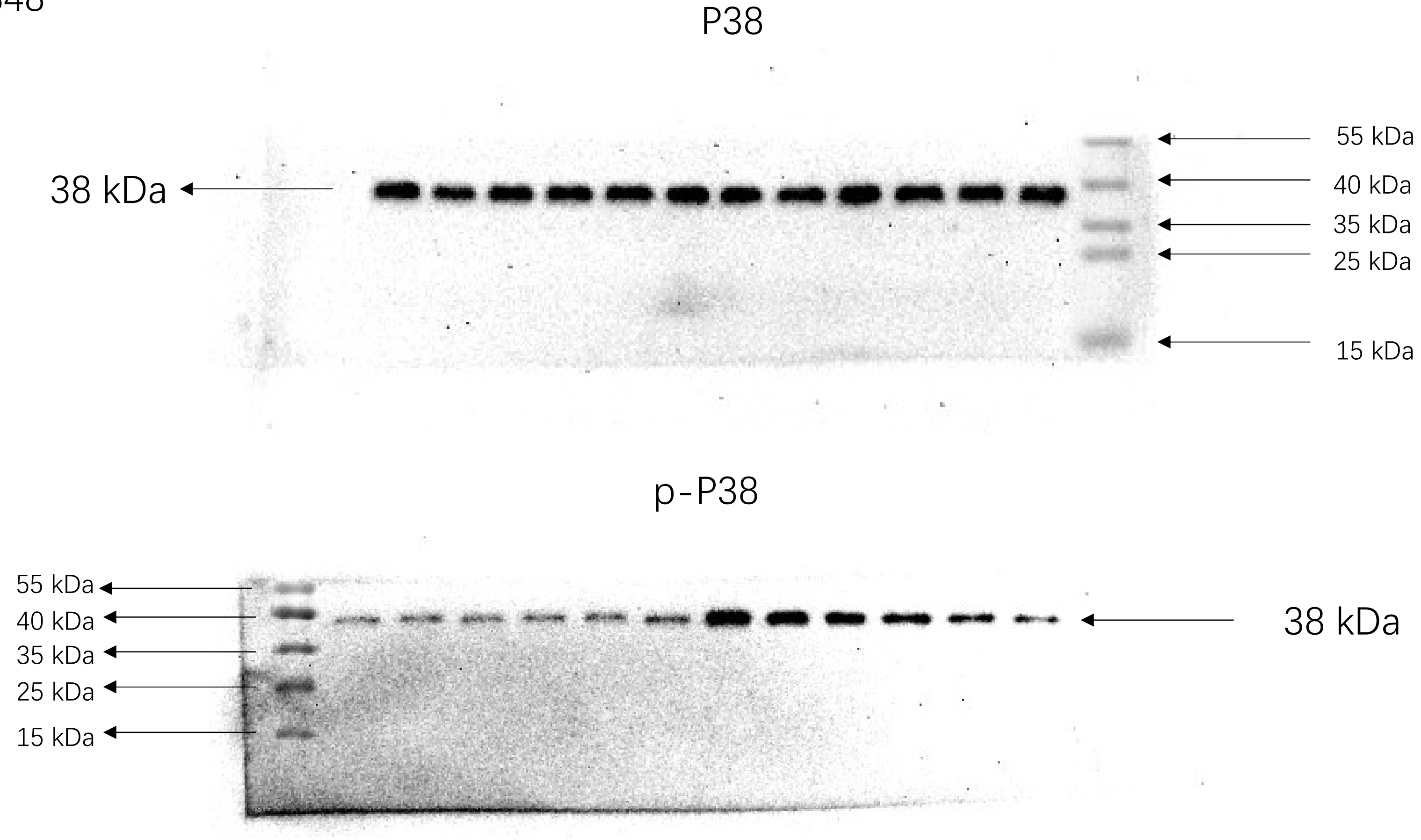

Fig 4 +AH23848

P65

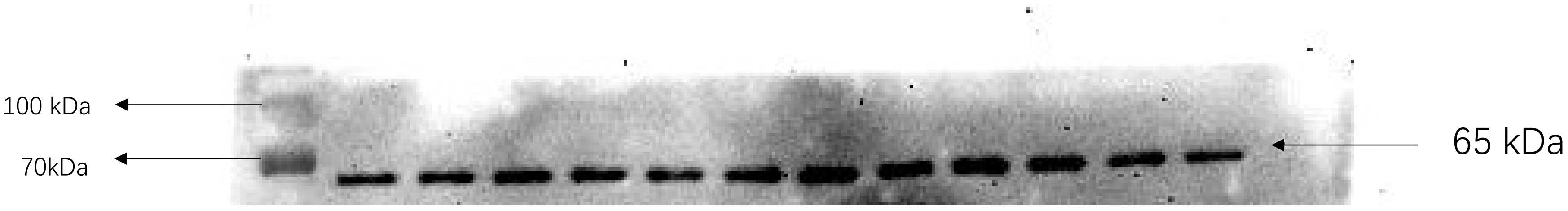

p-P65

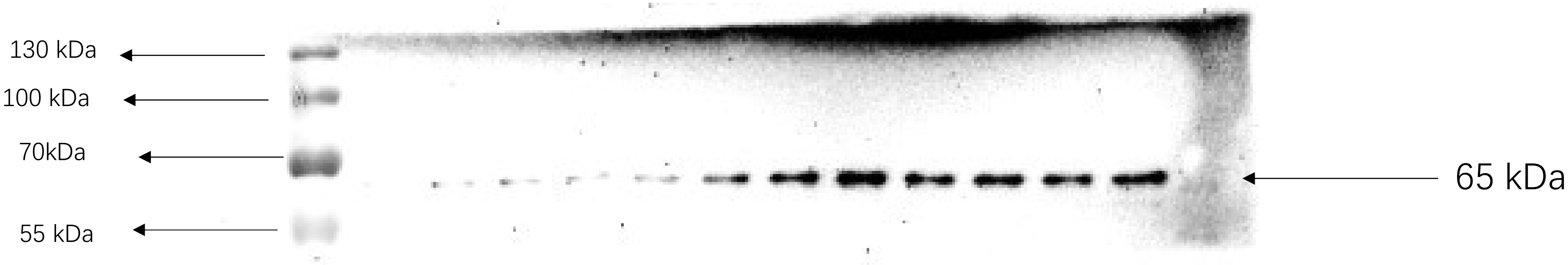

Fig 4 +AH23848

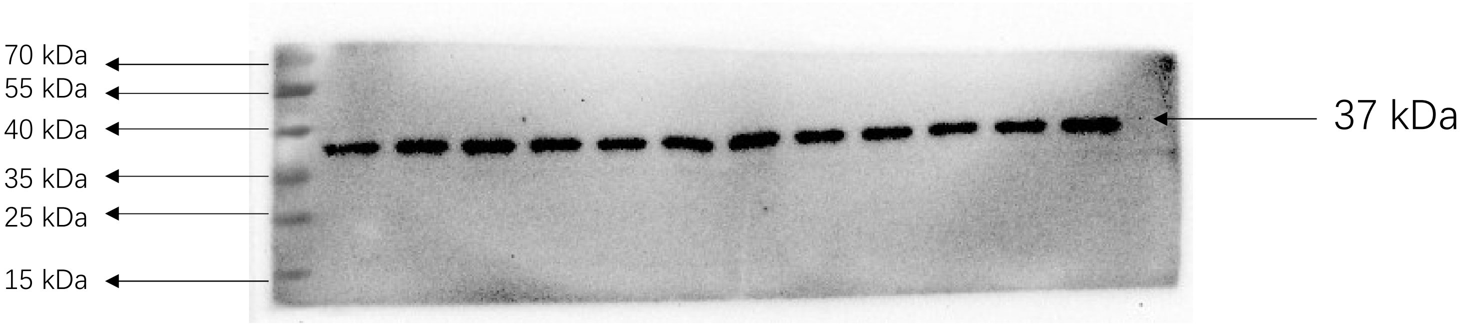

GAPDH
